# Biotechnological Advances to Improve Abiotic Stress Tolerance in Crops

**DOI:** 10.3390/ijms231912053

**Published:** 2022-10-10

**Authors:** Miguel Angel Villalobos-López, Analilia Arroyo-Becerra, Anareli Quintero-Jiménez, Gabriel Iturriaga

**Affiliations:** 1 Laboratorio de Genómica Funcional y Biotecnología de Plantas, Centro de Investigación en Biotecnología Aplicada, Instituto Politécnico Nacional, Ex-Hacienda San Juan Molino Carretera Estatal Km 1.5, Santa Inés-Tecuexcomac-Tepetitla 90700, Tlaxcala, Mexico; 2 División de Estudios de Posgrado e Investigación, Tecnológico Nacional de México/I.T. Roque, Km. 8 Carretera Celaya-Juventino Rosas, Roque, Celaya 38110, Guanajato, Mexico

**Keywords:** abiotic stress, osmoprotectants, drought tolerance, effective-microbes, genotyping-by-sequencing, NPBT, plant transformation, QTLs, site-directed mutagenesis, CRISPR, ZFNs

## Abstract

The major challenges that agriculture is facing in the twenty-first century are increasing droughts, water scarcity, flooding, poorer soils, and extreme temperatures due to climate change. However, most crops are not tolerant to extreme climatic environments. The aim in the near future, in a world with hunger and an increasing population, is to breed and/or engineer crops to tolerate abiotic stress with a higher yield. Some crop varieties display a certain degree of tolerance, which has been exploited by plant breeders to develop varieties that thrive under stress conditions. Moreover, a long list of genes involved in abiotic stress tolerance have been identified and characterized by molecular techniques and overexpressed individually in plant transformation experiments. Nevertheless, stress tolerance phenotypes are polygenetic traits, which current genomic tools are dissecting to exploit their use by accelerating genetic introgression using molecular markers or site-directed mutagenesis such as CRISPR-Cas9. In this review, we describe plant mechanisms to sense and tolerate adverse climate conditions and examine and discuss classic and new molecular tools to select and improve abiotic stress tolerance in major crops.

## 1. Introduction

Anthropogenic climate change is remodeling our planet due to an increase in gas emissions and deforestation, creating a greenhouse effect which is dangerously raising the Earth’s temperature. A wide list of consequences includes devastating hurricanes, scarce rainfall, migration, and extinction of different species of plants and animals due to the destruction of their habitats, and the appearance of diseases that affect all species, including humans. All this entails the depletion of natural resources, jeopardizing our survival and that of many species and ecosystems [1,2]. In fact, global warming is reshaping geographical species distribution, altering the composition of plant communities [3].

Agriculture and food security will suffer a significant impact due to climate change; therefore, new agricultural practices must cope with severe droughts, extreme temperatures, soil erosion and salinity, and devastating floods. In addition, there is an increasing freshwater scarcity in a world where the human population is growing exponentially and is estimated to account for almost 10 thousand million people in 2050. According to experts, food production should be double the current amount for that decade [4,5,6]. Therefore, a major technological effort, framed under a sustainable and ecologically sound world policy, is required to withstand such alarming, predicted conditions. Ultimately, there is a limit as to how far agriculture can adapt to the changing climate, and a political will to reduce the impact of the burning of fossil fuels on the global climate is essential for long-term food security [7].

### 1.1. Abiotic Stress Adaptations

**Figure 1:**
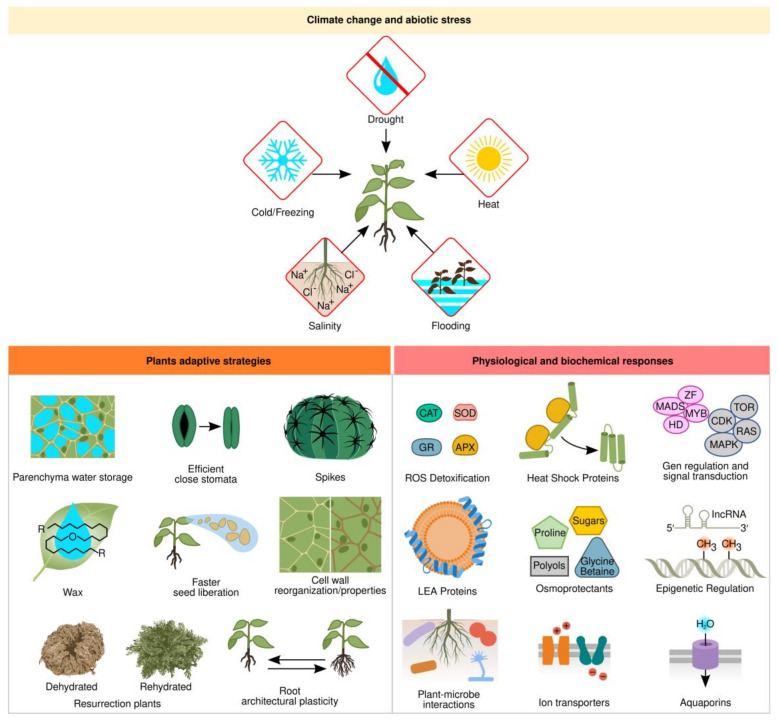
Plant response mechanisms to abiotic stress; Abbreviations: ROS—reactive oxygen species; CAT—catalase; GR—glutathione reductase; SOD—superoxide dismutase; APX—ascorbate peroxidase.

Plants often have to adapt to environments that are unfavorable for growth and development. Some plants acquired adaptive traits during evolution to deal with extreme environments, such as deserts, tundra, or swamps. To withstand these harsh environmental conditions, plants developed morphological and physiological adaptations, as well as signaling pathways that elicit biochemical and molecular mechanisms to survive different stress conditions [8]. The stressful environmental factors can be either biotic or abiotic. Abiotic stresses mainly include drought, salinity, extreme temperatures, flooding, oxidative stress, nutrient deficiencies, and heavy metal stress [9]. Drought is the major cause of crop losses around the world and water provision for agriculture was a key element for civilization’s success [10,11]. Drought, salinity, cold, and freezing induce osmotic and oxidative stress and increase intracellular ion concentration, thus leading to reactive oxygen species (ROS) accumulation, protein denaturation, membrane disruption, and nucleic acid damage (Figure 1).

At least three adaptive strategies have evolved in plants to live under drought conditions [12]. Ephemeral plants are annual and have a short life cycle that allows them to grow and develop during the rainy season before forming seeds to escape unfavorable conditions. Another survival strategy is present in Cactaceae and Agavaceae plant families, which due to the presence of spikes as modified leaves, a long root system to capture as much water as possible, their stem/leaves sheltered with a thick waxy cuticle that covers a water-storage parenchyma, and adjusting their photosynthesis system with closed stomata and a C_4_-carbon assimilation system, maintain the plant as a water reservoir to avoid water loss. The last group is true desiccation-tolerant plants, better known as resurrection plants, which can survive long periods of even decades without water to restart their metabolism and growth in a few hours after being again in contact with water (Figure 1) [13].

Plant desiccation-tolerance was a key trait to conquering land environments, although it is rare in vegetative tissues it is present in mature seeds but is lost during germination [14]. Some genetic and biochemical components for drought tolerance are shared between seeds and resurrection plants and to some extent in non-tolerant plants displaying hardening or acclimation phenotypes [15]. These latter plants require the expression of stress tolerance genes for a gradual adaptation to harsh conditions. Cultivated plant varieties are usually more sensitive to abiotic stress than their wild-type relatives because breeding selection for yield and plant size traits are not necessarily linked to stress tolerance genes.

A water deficit reduces photosynthesis by inducing stomata closure and inhibiting electron transport reactions and oxygen production, thus leading to ROS accumulation, which causes damage to the photosynthetic reaction centers [16]. Plant adaptations to photosynthesis damage under drought stress are limited to a few plants containing C_4_ carbon assimilation metabolism and crassulacean acid metabolism, which minimize photorespiration and a more efficient CO_2_ harvest [17]. Gene transfer experiments using transcription factors and Calvin-Cycle enzyme genes might soon improve photosynthesis and yield under abiotic stress conditions [18,19].

Another important adaptation to water stress involves root architectural plasticity (Figure 1). Plants with longer roots usually display increased yield since they have a higher water use efficiency measured as biomass production to water use ratio [20]. During drought, ABA signals the SNAC1 transcription factor, which induces stomata closure, shoot growth arrest, and induces root growth, whereas lateral root growth is reduced due to the expression of the *MYB96* transcription factor and microRNA miRNA393 [21,22].

### 1.2. Physiological and Biochemical Responses

Protective mechanisms against abiotic stress include osmotic adjustment, antioxidant metabolism, and maintenance of cell membrane stability (Figure 1) [23]. Plant cells actively accumulate solutes when cellular dehydration occurs as part of osmotic adjustment [24]. Metabolic-compatible compounds can accumulate in large quantities and are major drivers of osmotic adjustment under salt, drought, and cold stresses, helping with membrane stabilization, protection of the quaternary structure of proteins, and neutralization of toxic compounds under stressful conditions. It is the common strategy adopted by many organisms to combat environmental stress [25]. These compatible compounds, known as osmoprotectants or osmolytes, are low molecular weight water-soluble compounds. The most common but not exclusive compatible solutes are sugars (sucrose and trehalose), polyols (mannitol and sorbitol), polyamines (putrescine, spermine, and spermidine), amino acids (glutamine and proline), and quaternary amines (glycine-betaine and choline-O-sufate) [26]. For instance, spinach, sugar beet, and amaranth accumulate high levels of glycine-betaine, which is synthesized by two enzymatic steps. First, choline monooxygenase converts choline into betaine aldehyde, and then betaine aldehyde dehydrogenase synthesizes glycine-betaine [27]. These enzymes are found in the chloroplast stroma. Proline is an osmolyte with an active role in plant growth and development in many plants, such as maize, rice, and legumes, and is synthesized in the cytoplasm through two major pathways. In the glutamate pathway, it is produced from glutamate by Δ_1_-pyrroline-5-carboxylate synthetase and Δ_1_-pyrroline-5-carboxylate reductase enzymes; in the ornithine pathway by ornithine-delta-aminotransferase, producing glutamic semialdehyde and Δ_1_-pyrroline-5-carboxylate, which is converted to proline [28]. Another important class of compatible solutes is sugar alcohols, also known as polyols, found in a wide range of species such as grapevine, apple, coffee, berries, and olives. Sorbitol and mannitol are synthesized by specific polyol dehydrogenases either from glucose-6-phosphate or mannose-6-phosphate, respectively, and protect cells against osmotic stress and metabolic imbalance between source and sink plant organs [29].

A peculiar molecule is the disaccharide trehalose, which functions as an osmoprotectant against heat, salt, and drought stresses and is found at high concentrations in certain archaea and eubacteria species, some fungi and lower invertebrates, and desiccation-tolerant plants such as the lycophyte *Selaginella lepidophylla* and in some rare Poaceae family members [30]. There are five biosynthetic pathways, and the most common in several organisms, including plants, is a two-step process, where initially trehalose-6-phosphate synthase (TPS) condenses glucose-6-phosphate and UDP-glucose into trehalose-6-phosphate, which is converted to trehalose by trehalose-6-phosphate phosphatase (TPP). Interestingly, both TPS and TPP are also present in most plants and are encoded by multi-gene families; however, trehalose is almost undetectable [31]. The intermediate compound trehalose-6-phosphate also acts as a signal molecule involved in growth, development, and crop yield [32,33].

The non-toxic nitrogenous polycationic molecules named polyamines, usually bound to nucleic acids and proteins, accumulate in all organisms, and are involved in membrane stability and ROS scavenging [34]. Some authors consider polyamines as phytohormones since they participate in various plant functions, including regulating cell division, membrane and cell wall stabilization, growth, flower and fruit development, and adaptation to biotic and abiotic stresses [35].

Oligosaccharides such as fructans, staquiose, and raffinose are involved in cold acclimation and freezing tolerance in a wide range of plants [36]. For instance, in spite of lacking epidermal tissues, *Physcomirium patens* moss displays freezing tolerance due to the accumulation of the trisaccharide theanderose [37].

Major cultivated crops such as wheat, rice, and maize do not accumulate osmolytes at significant concentrations [27]. However, it has been shown that the overexpression of proline, glycine-betaine, fructans, and trehalose biosynthetic genes leads to abiotic stress-tolerant phenotypes and plants with higher biomass and yield [38]. In addition to their role as osmolytes, all these compounds are also involved in inhibiting ROS accumulation and protecting the photosynthetic apparatus.

Similar to osmotic adjustment, antioxidant defense systems are also important to stress tolerance mechanisms (Figure 1). Drought, cold, heat, and salt stress induce the formation of reactive oxygen species (ROS) such as hydrogen peroxide (H_2_O_2_) and superoxide (O_2_^−^) [39]. These ROS are extremely toxic compounds, which can severely damage cell membranes, proteins, and DNA, eventually provoking cell death. In order to combat oxidative damage, plants utilize antioxidant defense mechanisms including enzymes such as superoxide dismutase (SOD), ascorbate peroxidase (APX), peroxidase (POD), catalase (CAT), and glutathione reductase (GR) [40] (Figure 1). In this regard, sugars also act as ROS quenchers and exert protective roles and stress tolerance when specific sugar types are localized in specific cellular compartments or in close vicinity membranes [41]. Crops with enhanced ascorbate accumulation promote abiotic stress tolerance either by engineering increased biosynthesis, enhanced recycling, or modulating regulatory factors [42].

Heat during summer days and at midday is a major stress that plants cope with by accumulating heat-shock proteins (HSP) to alleviate cellular damage (Figure 1). HSPs are a broad protein family of molecular chaperones that comprises HSP100, HSP90, HSP70, HSP60, HSP40, and HSP20 that prevent and restore protein aggregation and misfolding and are present in bacteria and animals as well [43]. Overexpression of different HSPs can partially alleviate heat stress in plants; however, engineered expression of upstream elements in the signaling pathway, as exemplified by the HSP transcription factor (HSF), which confers a better response, also induces tolerance to multiple stresses [44].

As an additional tool to cope with abiotic stress, plants acquired a group of low molecular weight (10–30 kDa) proteins to protect against subcellular damage caused by drought, salt, or cold stress. These are named late embryogenesis abundant (LEA) proteins rich in glycine and lysine and have a hydrophilic and disordered nature, which under stress conditions reorders and forms repeated α-helixed stretches (Figure 1) [45]. LEAs were originally found in mature seed embryos but, later on, were also identified in stressed vegetative tissues of most plants and even in some desiccation-tolerant invertebrates [14,46]. They are classified into five families according to their protein similarity and are localized in the nuclei and cytoplasm, interacting with cellular proteins to prevent unfolding or stabilize membranes during osmotic stress [45]. Either an ABA-dependent or an ABA-independent pathway can induce *LEA* genes. For instance, two well-studied cases in Arabidopsis are the *RD29A* and *RD29B* LEAs protein genes. The *RD29B* gene promoter has an ABA-regulated element, the ABRE box (CACGTG), whereas the *RD29A* promoter has an ABA-independent dehydration-responsive element, the DRE box (TACCGACAT), induced by desiccation salt and cold stresses [47]. The overexpression of the pepper CaLEA1 in Arabidopsis confers osmotic stress tolerance [48].

Water transport along the plant from the soil into the roots is a key element for plant development and physiology (Figure 1). Aquaporins function by regulating water trafficking through cell membranes in normal and stressful conditions but can also transport small solutes such as monosaccharides, polyols, silicon, and boron or gases such as ammonia or carbon dioxide [49]. There are between 25 and 30 kDa proteins forming transmembrane tetramers and belonging to the major intrinsic protein (MIP) superfamily with members in most organisms [50]. In plants, aquaporins are classified into seven subfamilies according to their sequence similarity, most of them plasma membrane intrinsic proteins (PIPs), tonoplast intrinsic proteins (TIPs), and nodulin 26-like intrinsic proteins (NIPs) [51].

Together with drought, soil salinity is severely threatening agriculture since around 20% of total cultivated land and 50% of irrigated fields worldwide are affected by salinization [52]. Salt stress inhibits plant growth and yield, germination, photosynthesis, and transpiration, and alters ion homeostasis (K^+^/Na^+^) caused by the combined effects of osmotic stress and ion toxicity, mainly due to Na^+^ and Cl^−^ accumulation in the plant cytosol (Figure 1) [53]. Plant osmotic stress is dealt with the aid of osmoprotectants, and antioxidant molecules previously discussed. According to their response to salt stress, plants are classified as salt-tolerant or halophytes, and salt-sensitive or glycophytes [54]. When Na^+^ levels rise excessively in the plant, the K^+^ uptake is inhibited, the latter being an essential ion for photosynthesis and metabolism as an enzyme cofactor. Hence, plant cells need to either extrude Na^+^ from the cell, accumulate it on the vacuole, which also maintains cell turgor, or transport Na^+^ to a specialized organ such as the trichome in some halophytes [53]. Salt traffic requires transporters in plasma or vacuole membranes. Several transporter genes have been characterized [55]. The plasma membrane symporters HKT1 in leaves and ATK1 in roots are responsible for pumping K^+^ into the cell. Another significant pump is the Salt Overly Sensitive (SOS1) antiporter, which extrudes Na^+^ and transports H^+^ into the cell [56]. SOS1 is regulated by phosphorylation of SOS2 protein kinase and Ca^++^ sensor SOS3. On the vacuole membrane, Ca^+^ regulates Na^+^ compartmentalization by the NHX1 antiporter and helps H^+^ exit. The other key types of antiporters are V-ATPase and V-PPase, which are responsible for introducing H^+^ into the vacuolar membrane [55].

### 1.3. Gene Regulation and Signal Transduction

Complex signaling cascades are induced in response to abiotic stress and are integrated into multistep phosphorelay signaling that includes both hormonal and environmental cues into a common pathway [57]. The different abiotic stresses can trigger common signaling pathways [58]. Plant sensing to abiotic stress initiates with membrane or intracellular osmosensors that induce a shift in intracellular Ca^2+^ and production of secondary messengers such as inositol phosphate (IP) and ROS, leading to activation of different protein kinases such as calcium-dependent protein kinases (CDPKs), calcium/calmodulin-dependent protein kinases (CCaMKs), mitogen-activated protein kinases (MAPK), or phosphatases that can phosphorylate/dephosphorylate specific transcription factors, which in turn regulate stress-responsive genes [59]. There is an intricate crosstalk among various plant hormones, namely, abscisic acid (ABA), jasmonic acid (JA), salicylic acid (SA), cytokinins (CK), and ethylene (ET), in coordination with transcription factors such as MYB, bLHL, WRKY, bZIP, NAC, and DREB, to activate or repress stress responses [60] (Figure 1).

ABA is a major player in coordinating plant stress responses. Several abiotic stresses, such as drought and high salinity, trigger ABA biosynthesis, which is responsible for key plant processes such as root growth, stomatal closure, and seed germination. The core regulatory pathway of ABA signaling has been identified [58]. ABA interacts with the regulatory component of the ABA receptor, which in turn inhibits phosphatase type 2C (PP2C), promoting the liberation of SNF1-related protein kinase 2 (SnRK2). The activated SnRK2s phosphorylate downstream effectors to regulate multiple biological processes, such as transcription, RNA processing, epigenetic modification, and flowering time regulation [61].

JA, a class of fatty acid-derived molecules, also plays an important role in promoting plant response to abiotic stress. It participates in several signal transduction pathways to induce cell protection from the toxic effects of salt stress, drought stress, heavy metal toxicity, micronutrient toxicity, freezing stress, ozone stress, and light stress [62].

CK acts as a negative regulator of salt and drought tolerance and counteracts ABA and Strigolactones (SLs), a carotenoid-derived phytohormone, which has roles in root development, shoot branching, leaf senescence, plant communication with beneficial microbes, and adaptation to cold, saline, and drought stresses [63].

Several other molecules are novel plant growth regulators, acting in signaling cascades and cross-talk with regulatory machinery. The precursor of heterocyclic compounds such as chlorophyll, 5-aminolevulinic acid (ALA), improves abiotic stress tolerance, growth, and yield by inducing antioxidant synthesis, nutrient uptake, and photosynthesis [64]. Citric acid/citrate (CA) has similar effects and relieves heavy metal stress by inducing chelation and precipitation of metal ions [65]. Nitric oxide (NO) is a redox-active gas that at low concentrations can signal abiotic stress responses in plants by interacting with calcium and hydrogen sulphide [66]. Melatonin works as an antioxidant against ROS to promote photosynthesis, rooting, growth, and biotic and abiotic stress tolerance, and its membrane receptor has been identified [67]. Recent studies have revealed the presence of hormone-like peptides and their corresponding receptors, involved in signaling tolerance to abiotic stress [68]. Interestingly, the SCREW-NUT peptide-receptor pair counteracts the ABA- and pathogen-induced stomata closure [69].

High-throughput sequencing has revealed an extensive number of miRNAs in plant genomes as key regulators in plant development and mediating biotic and abiotic stress responses [70]. Outstandingly, another emerging area of stress tolerance is epigenetics, which deals with genetic changes in chromatin functions that are not related to changes in DNA sequence (Figure 1). Epigenetic modifications, such as DNA methylation and histone acetylation or phosphorylation, prepare chromatin accessibility for transcription machinery, inducing different chromatin conformations for stress-responsive gene expression [71]. In addition to DNA and histone modifications, multiple long non-coding RNAs (lncRNAs) are the top emerging participants in abiotic stress responses, regulating transcription factors, numerous miRNAs, and stress-responsive mRNAs [72]. The active changes in epigenetic modifications on stress-responsive genes open or close the chromatin accession to transcriptional or posttranscriptional regulatory machinery. For instance, HDA6 deacetylase is a negative regulator of the PDC1 and ALDH2B7 genes and thus represses acetate biosynthesis. Under drought, the HDA6 enzyme dissociates from the PDC1 and ALDH2B7 genes to activate the acetate pathway. Acetate connects the plant’s ability to adapt to drought stress with metabolism, epigenetic regulation, and JA signaling [73].

### 1.4. Effective Microbes

**Figure 2:**
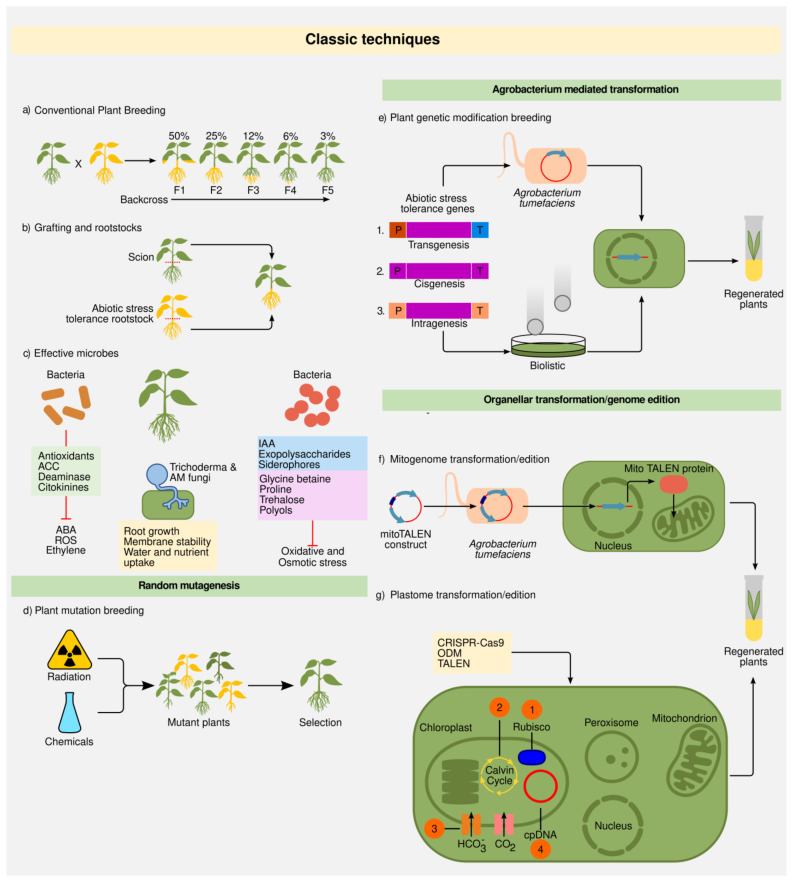
Crop breeding classic techniques and molecular tools. (**a**) Conventional plant breeding. Pollen from a plant with a desired trait is transferred to the flowers of another variety with other desirable characteristic. Eventually, the desired trait(s) will appear in a new variety of plants through selection. (**b**) Grafting and roostooks. Grafting is a special type of asexual plant propagation, where a section of a plant (scion) is joined to another plant (rootstock), allowing it to grow as a single plant sharing a unified vascular system. To combine two groups of desired characteristics, usually, both scion and rootstock sources become from different plant varieties. (**c**) Effective microbes. Plant and microbe interactions involve highly sophisticated symbioses that confer stress tolerance. PGPRs can produce antioxidants, 1-aminocyclopropane-1-carboxylic acid (ACC) deaminase, cytokines, auxine indoleacetic acid (IAA), exopolysaccharides, siderophores, that inhibit absicic acid (ABA), reactive oxygen species (ROS), and ethylene negative effects. Some bacteria can also produce compounds to increase the solubility and uptake of nutrients from soil or synthetize osmoprotectans that can improve drought responses to plants. Additionally, some fungi help plants by increasing water and nutrient uptake. (**d**) Plant mutation breeding. It is induced by physical stimulations (X-rays, α and β particles, fast neutrons, and ultraviolet light), or chemical treatments (ethyl methanesulfonate) that generate chromosomal changes that cause random mutations. (**e**) *Agrobacterium tumefaciens* and biolistic mediated plant genetic modification breeding. P: promotor; T: teminator. (1) Transgenesis: one or more components such as gene, P, and T that come from sexually incompatible organisms (2) Cisgenesis: all components come from the same original gene (P, gene, and T) isolated from the same species or a sexually compatible organism; (3) Intragenesis: uses one or more components (such as gene, P or T) from different genes of the same species or a sexually compatible organism. (**f**) Mitogenome transformation/edition. It is carried out through transcription activator-like editing nucleases (TALEN) mediated nuclear transformation. (**g**) Plastome (plastid genome) transformation/edition. Chloroplast transformation has been used for: (1) Improvement of the catalytic activity of the RUBISCO enzyme, (2) maximize carbon fixation (Calvin cycle) (3) insertion of cyanobacteria transporters, (4) edition focused on different components related to the DNA damage response of the genome, among other applications.

Rhizosphere microorganisms such as bacteria and fungi promote plant growth and yield but also have an important role in abiotic stress tolerance enhancement in plants (Figure 1 and Figure 2c). Plant-microbe interactions signal molecular networks that modulate phytohormone status and gene expression in plants, inducing osmolyte and nutrient accumulation, antioxidant and proton transport machinery, and ion compartmentalization that elicits stress-responsive pathways [74,75,76]. Plant growth-promoting rhizobacteria (PGPR) can synthesize phytohormones such as indole-3-acetic acid (IAA), an auxin that induces root and shoot growth, or cytokinins that promote cell division and differentiation [77]. PGPR can also induce plant accumulation of ABA, enhancing the expression of drought tolerance genes that render osmoprotectants and K^+^ accumulation, decrease in electrolyte leakage, and increase ROS scavenging capacity [78]. Plant inoculation with PGPR that contains 1-Aminocycloprpane-1-Carboxylate (ACC) deaminase reduces ethylene and promotes plant growth and yield under salt stress conditions [79]. Siderophore produced by PGBR facilitates atmospheric nitrogen fixation, phosphate solubilization, and helps nutrient mobilization [80]. Exopolysaccharides (EPS) from PGPR aid in the formation of biofilms to keep soil moisture and protect plant roots under water scarcity conditions [81]. Another important group of soil microorganisms that interact with plants are Trichoderma and arbuscular mycorrhizal (AM) fungi that also mitigate abiotic stress by promoting root growth, membrane stability, and water and nutrient uptake [82,83]. Through triggering phytohormone signaling, these fungi activate plant aquaporins and membrane transporters gene-expression, improve photosynthesis by scavenging ROS, and induce osmolyte accumulation in plant cells to maintain water use efficiency under stress conditions [83].

*Trichoderma atroviride* inoculation diminishes drought effects in maize seedlings by inducing antioxidant machinery [84]. Moreover, combined inoculation with *Trichoderma* and *Pseudomonas* minimized the impact of a low watering regime in rice and upregulated genes linked with enzymatic and non-enzymatic antioxidant reactions that helped plant survival under stress [85]. Recently, a collection of *Trichoderma harzianum* mutants obtained by mutagenesis with ethyl methanesulfonate enhanced drought tolerance in Jalapeño pepper plants, phosphorus assimilation, and antagonism against phytopathogenic fungi [86]. Thus, all these results emphasize that the inoculation of growing plants with microorganism formulations, also known as biofertilizers, has a strategic potential in cultivating crops under abiotic stress conditions.

## 2. Molecular Tools

### 2.1. Classic Techniques

#### 2.1.1. Plant Breeding

The need for more food production and the intrinsic curiosity of man culminated with the domestication of important crops (about 1200 years ago) and the application of empiric plant breeding processes by the selection of desired traits [87]. However, only 200 hundred years ago, plant breeding started to be used in a systematic way to improve yields and to select the presence of desired characteristics [88]. The Mendel laws provided the basic rules for starting the breeding of crops, but it was only until the early twentieth century that the first hybrids were developed that improved agriculture’s yield. An important innovative process for plant breeding was the Green Revolution in the 1960s, developed by Nobel laureate Norman Borlaug, which significantly increased agricultural production by breeding new high-yielding cereals such as dwarf wheat and rice varieties used in combination with chemical fertilizers, pesticides, mechanical irrigation, and machinery.

The generation of new varieties by conventional plant breeding requires either selecting plants with desirable traits or combining qualities from two closely related plants through selective breeding [89]. Pollen from a plant with a desired trait is transferred to the flowers of other varieties with a new advantageous characteristic. Eventually, the desired trait(s) will appear in a new variety of plants through offspring selection. However, this is not an easy task because DNA from the parents recombines randomly, and the desired traits may be linked with undesirable traits. Traditional strategies to insert a desired characteristic in a first-class cultivar involve the introgression of associated alleles across at least six generations of consecutive selection. For instance, the insertion of an abiotic stress-resistance trait into a high-yielding cultivar begins with their cross with an abiotic stress-resistant cultivar. Then, recurrent backcrossing with the elite ancestor is required to maintain the genetic potential of the elite cultivar besides preserving the recently imported resistance allele (Figure 2a). Typically, the entire procedure requires several generations to fix the resistance allele in the elite background [90]. Thus, the required time to release a new crop variety takes on average 12–15 years [91], depending on the crop.

Plant breeding involves the following two key steps: the generation or expansion of new variations and the selection and fixation of desirable genotypes [87]. Methods such as hybridization, induced mutation, chromosome manipulation, F1 hybrids, and the transgenic approach, among others, have been used to create variation. Initially, the selection was supported essentially by the phenotypic appraisal of target traits, easily distinguishable by the eye. More recently, with the advent of modern selection methods, traceability of hidden traits has become possible. On the other hand, for selection and generation advancement, methods such as simple phenotypic selection, recurrent selection, marker-assisted selection (MAS), genomic selection, and heritability and genetic gain after selection are used, among others.

Modern breeding for major crops requires the use of molecular markers, where DNA markers such as RFLP, SSR, AFLP, RAPD, or SNP are the most frequently used [92]. However, to develop trait-linked markers, segregating populations for genotyping by sequencing the target traits and reliable phenotyping methods are indispensable [93]. Molecular markers can be enforced to marker-assisted selection (MAS), which capitalizes on the genetic correlation between target gene(s) and phenotypes. An important condition prior to MAS is the construction of a molecular genetic map and also the detection of the target trait-linked markers. It is calculated that plant breeders working with conventional phenotypic selection should test from 1.0 to 16.7 times more breeding lines in comparison to breeders adopting MAS. To test such a number of lines is important to ensure the introduction of one or more superior genotypes, relying on genotypic superiority, selection pressure, and heritability level of traits. Hence, MAS can significantly decrease the time and resources required to accomplish a selection goal for heritability traits of low to moderate values when the selection intensity is high [94].

Essential agronomic features, such as stress tolerance and crop yield, are governed by polygenes, which exert refined effects and are regulated by the environment. Such characteristics are known as quantitative traits, and the segregating loci as quantitative trait loci (QTLs). In the case of quantitative traits, the fundamental procedure is to detect markers linked to the quantitative trait via QTL [92,95,96,97,98]. Moreover, many important QTLs from crop species have been cloned thanks to the increasing availability of whole genome sequences. Therefore, QTL mapping surged as single-marker mapping but has now become interval mapping thanks to the use of multiple marker-based approaches. The accumulated information related to QTLs is available from several public databases, and a robust compilation of available databases of genomic resources for plant breeding was recently published [88].

Crop phenomics constitutes a new area of development for plant breeding, with constant technical innovations arriving. These high-throughput phenotypic technologies are crucial tools to accelerate the advancement of genetic gain in breeding programs. The massive phenotypic data collection through diverse sensors (crop morphology, structure, and physiological status from cell to the whole plant) is an important part of crop phenotyping, although the statistical integration of such data (phenomics analysis) is an important challenge for the optimization of this strategy [99].

#### 2.1.2. Grafting and Rootstocks

Plant grafting is one of the most valuable tools against soil-borne diseases and biotic or abiotic stress factors. Grafting allows for higher yields under stressful conditions, an extension of the cultivation period, lower use of fertilizers and agrochemicals, broad use of phytogenetic resources as rootstocks, and no need for crop rotation [99,100,101,102,103].

Grafting is a special type of asexual plant propagation where a section of a plant (scion) is joined to another plant (rootstock), allowing it to grow as a single plant sharing a unified vascular system [104]. In order to combine two groups of desired characteristics, usually both scion and rootstock sources are from different plant varieties. The re-establishment of the new plant entity starts with tissue connection between the rootstock and the scion at the grafting points, it continues with a dynamic cell division phase that results in the development of a callus and common cell wall, and it ends with the establishment of a unique vasculature system [103,105] (Figure 2b).

Important fruit crops are frequently propagated by grafting methods, such as apples, avocados, peaches, citrus, apricots, cherries, plums, and almonds [104,106], and also conifers [107], among others. Only a few decades ago, vegetable crops were incorporated into the grafting practice [101,103,108,109]. Depending on the required situation, seed companies and breeders have used different rootstocks with the appropriate characteristics [110,111]. The trans-grafting method mixes classical grafting protocols with the genetic engineering of plants. The trans-grafting method comprises the grafting of a non-genetically modified scion onto a genetically altered rootstock. The scion obtains benefits and traits conferred by transgenes in the rootstock, but the end products, such as fruits, do not contain the transgene and hence are not genetically modified [112,113]. Another innovation is micrografting, which involves the in vitro grafting of small shoot apices or lateral buds onto decapitated rootstock seedlings [110,114,115].

The molecular basis of grafting signaling is not fully understood, but recent research suggests that different plant hormones, proteins, epigenetic events, and several types of RNA could be responsible for changes in scion [103,113,115,116,117,118,119,120].

#### 2.1.3. Random Mutations

Random mutagenesis is an essential tool to enhance crop diversity. The use of random mutagenesis has been widely accepted; besides that, it has no regulatory restrictions. Induced mutations can be generated by the use of physical mutagens such as X-rays, gamma rays (cobalt-60 being the most common source of radiation), and neutrons [121,122,123,124,125,126,127]. Moreover, to induce mutagenesis, chemical mutagens, including alkylating agents such as ethyl methanesulfonate (EMS), intercalating agents (such as ethidium bromide), and base analogs (such as bromouracil), can be used [121,128,129]. Nevertheless, physical mutagens are used more frequently as compared to chemical mutagens. Chemical agents, such as EMS, may result in more frequent mutations, but an important advantage of radiation over chemical mutagenesis is the fact that it induces a higher proportion of mutations that substantially affect gene functions (e.g., the deletion of a complete gene), thus resulting in more loss-of-function mutations related to target traits with fewer mutations per genome [125,130,131]. For both physical and chemical mutagenesis, the most utilized plant materials are their propagules (such as seeds and meristematic cells), tissues, and organs (cuttings, pollen, tissue-cultured calli), and in some cases, whole plants are also exposed [122] (Figure 2d).

Mutation induction is a powerful tool for creating new and novel plant germplasm [132]. Radiation-induced mutation, also known as plant mutation breeding, is the most widely used method to improve direct mutant varieties in a faster way in comparison with the laborious and time-consuming traditional plant breeding [125,127,133].

In vitro selection for plant mutation breeding can be used to obtain plant genotypes with tolerance to adverse environmental biotic or abiotic factors such as drought, high salinity, or even pathogens, in accordance with the selective media used [121,122,134]. Biological materials exposed to mutagens can be trustworthy and easily screened in a comparatively small space under in vitro conditions, in comparison to the use of a greenhouse and field. Purified lines of interest can be massively propagated or used for breeding purposes (Figure 2d).

#### 2.1.4. Plant Transformation: Biolistics and Agrobacterium

Plant genetic transformation refers to the generation of transgenic plants (transgenics), which harbor extra, stably integrated, and expressed foreign gene(s) generally from trans species (Figure 2e) [135,136]. These modified plants are generally named genetically modified organisms (GMOs). The complete process comprising the introduction, integration, and expression of foreign gene(s) in the host is termed genetic transformation or transgenesis [137]. Together, the development and application of molecular genetic engineering, gene transfer methods, and in vitro tissue culture techniques have allowed for the efficient genetic transformation of a broad diversity of crop plants [138,139].

Introducing DNA into plant cells is a key point for the generation of stable transgenic plants as well as transient transformation. Various gene transfer methods, such as the Agrobacterium system, physicochemical uptake of DNA, liposome encapsulation, protoplast electroporation, microinjection, DNA injection into intact plants, incubation of seeds with DNA, pollen tube pathway, the use of laser microbeam, electroporation into tissues/embryos, silicon carbide fiber method, particle bombardment, and “in planta” transformation, have been developed [139,140]. Among these, Agrobacterium and biolistic methods have been widely used for plant genetic transformation due to their efficiency and handiness [141,142,143,144,145,146,147] (Figure 2e). However, the transformation efficiency varies according to the plant species and cultivar.

Biolistic transformation (also called particle bombardment) consists of forcing DNA molecules into plant cells using high pressure as a physical medium. Contrasting with plant transformation mediated by Agrobacterium tumefaciens, plant transformation mediated by biolistic does not rely on host genotype or receptivity. The first successful particle bombardment system for plant cells was established in onion [148] and was quickly followed in multiple models and other recalcitrant crop species such as wheat, rice, and maize [149]. The integration of exogenous DNA into the genome arises by both non-homologous and homologous recombination [150]. Particle bombardment-mediated transformation is the most preferred method in experiments that demand rapid analysis and transient expressions, such as promoter analysis, protein localization, transcription factor characterization, pathway elucidation, hormonal regulation of genes, and promoter component identification [146]. Particle bombardment can deliver the preferred DNA into both nuclear and organellar genomes. For instance, chloroplasts in higher plants and chloroplasts as well as mitochondria in algae were successfully transformed by the biolistic genetic transformation approach [151,152,153,154,155]. Another difference between both methods for transformation is that Agrobacterium-mediated transformation can integrate from one to three copies of donor DNA, while the biolistic method can integrate multiple copies [156].

The biolistic machinery requires gene gun equipment attached to a helium tank filled at high pressure. DNA coated onto the gold or tungsten carriers (microprojectile) is shot into the recipient target tissue at high velocity by a gene gun. The microprojectile passes through the cell, while the coated DNA stays within the cell [155]. In addition to successfully delivering tungsten/gold particles coated with DNA, particle bombardment is also used to deliver whole viral particles, RNA molecules, and even bacterial cells such as A. tumefaciens.

In contrast with traditional breeding, plant transformation techniques introduce only the isolated gene(s) of agricultural relevance, the 25 bp T-DNA borders (if Agrobacterium is used), and a selectable marker, without co-transfer of unwanted genes from the donor. However, if the selectable marker is co-transformed into a separate plasmid, both genes can be segregated after back-crossing [157,158]. In addition, homologous sequences to T-DNA are naturally present in many plant species without any altered phenotype, including edible plants such as sweet potatoes [159,160]. As a consequence, transgenesis has arisen as a supplementary tool to perform single-gene breeding or transgenic breeding of crops. The recipient genotype is minimally disturbed, only to the lowest degree, which eliminates the need for repeated backcrosses [144]. GMOs have been created using foreign genes from diverse sources, such as viruses, bacteria, fungi, insects, humans, and other animals, and from unrelated plants. More recently, DNA obtained by chemical synthesis has also been introduced to plant cells.

Among public opinion, there is a certain rejection of the use of transgenics, alluding to the fact of using genes from distant species that normally could not carry out a sexual cross. GMOs social debate, regulation, and risk assessment are out of the scope of the present review ([161,162,163,164,165] for further reading). About this concern, there are alternatives to traditional transgeneses, such as intragenesis and cisgenesis [166] (Figure 2e). Both strategies use genetic material from the same species that is going to be transformed or from very close genetic pools or varieties. In addition, plants modified by intragenesis or cisgenesis must not contain sequences such as selectable marker genes or carrier vector fragments. The difference between both strategies lies in the fact that cisgenesis respects the genetic context of the gene to be introduced (promoter, exons, introns, and terminator), while intragenesis allows the use of promoters, exons, introns, and terminators from different genes of the same species.

Different molecular tools have been developed to facilitate the design and subsequent construction of the desired transgene or synthetic gene. Starting from restriction enzymes or the gateway system to newer protocols such as the one-step isothermal in vitro recombination that allows the enzymatic assembly of DNA molecules of up to several hundred kilobases [167]. Other toolkits have been built, such as the GoldenGate or the GoldenBraid systems [168,169,170], which are strategies based on the use of IIS-type restriction enzymes and on offering a wide variety of promoters, terminators, markers, or reporters made from reusable pieces of standardized DNA. Synthetic genes constructed in this way can be used to promote constitutive or inducible expression, gene silencing, protein-protein interactions, or expression of multiple genes, among other applications.

The cellular totipotency in the plant kingdom and the ability of A. tumefaciens (a gram-negative soilborne bacterium) to infect plant cells and to modify them at the genetic level have been combined to open up a new field in plant biotechnology, transgenic plant biology [143,171,172]. The identification of the bacterium A. tumefasciens as the causal agent of the generation of tumors in the tissues of various plant species marked the beginning of the study of the biology of Agrobacterium. Further studies identified an A. tumefaciens plasmid (Ti plasmid) as responsible for the tumor-inducing process, which contains all the necessary genes to infect and transfer the genetic information to plant cells. Afterward, the transferred DNA (T-DNA) region was identified in a plasmid (Ti plasmid). The T-DNA is not only directed and integrated into the recipient genome, but its opines and cytokinin genes are also transcriptionally expressed at high levels in the plant cells, these latter genes being responsible for the crown-gall phenotype. It was also established that the T-DNA fragment harbors specific 25-bp long signals, known as RB (right border) and LB (left border). Both RB and LB are the key elements for DNA transfer and thus the genes within them are dispensable and can be removed to yield a ‘disarmed’ vector leading to transformed plants without tumors. The development of transgenic plants using A. tumefaciens was conducted in tobacco by several independent research groups [173,174,175]. A valuable tool for plant transformation was the construction of the pBIN19 binary vector, which has been widely used [176,177]. Since then, an increasing number of important crops and a great variety of plant species have been genetically transformed by using A. tumefaciens [178]. Depending on the plant species, different explant sources may be used to be transformed [139,144,179].

### 2.2. New Plant Breeding Techniques (NPBT)

#### 2.2.1. Genotyping-by-Sequencing (GBS) and “Omics”

**Figure 3:**
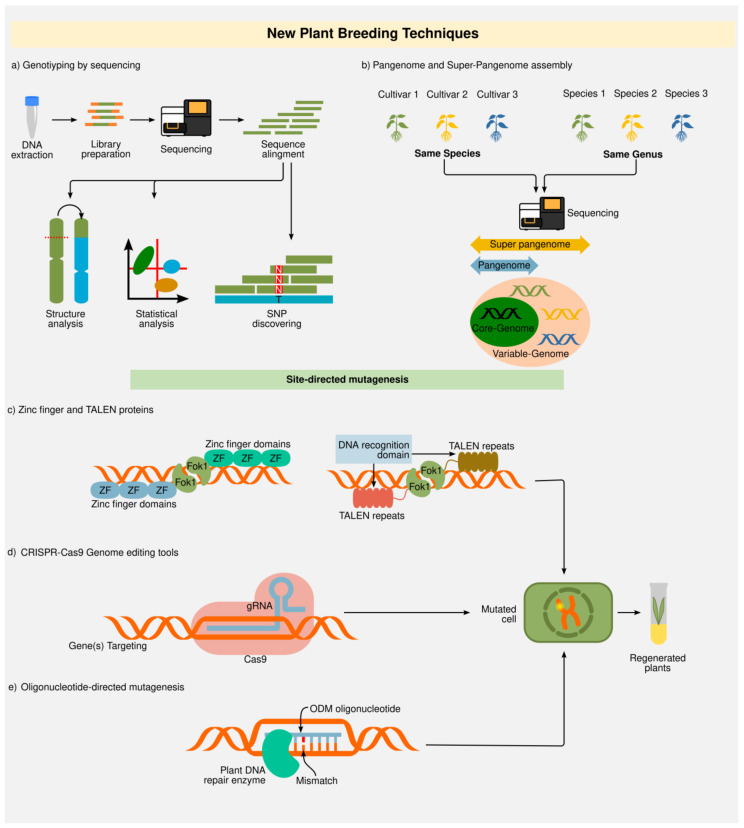
New plant breeding techniques. (**a**) Genotyping by sequencing. This method allows simultaneous analysis of large numbers of DNA samples using restriction enzymes to decrease genome complexity and generate thousads of SNP-like markers (one-base polymorphisms) using sequencing platforms. (**b**) Pangenome and super pangenome assembly. Describes the collection of all genes in a species (pangenome) or genus (super pangenome). These have a nucleus genome that has all the genes shared by a species and the variable genome that only some individuals have. (**c**) Zinc finger and TALEN site direct mutagenesis. Zinc finger nucleases are constituted by the fusion of series of zinc finger domains with a non-specific domain of the FokI nuclease. Each zinc finger domain recognizes three nucleotides while the FokI domain cuts DNA strands at different positions, introducing a sticky double strand break (DSB) (5–7 bp). Transcription activator-like effector nucleases (TALENs) are TALE repeats with a non-specific FokI endonuclease domain. Each TALE repeat recognizes a single nucleotide, while the FokI domain introduces sticky DSB within the spacer sequence (12–21 bp). (**d**) CRISPR-Cas9 genome editing tools. The system consists of a single guide RNA and Cas9 endonuclease. Guide RNA pairs with 20-nucleotide target sequences in genomic DNA, and the Cas9 enzyme contains catalytic nuclease domains that allow for site-specific editing. (**e**) This technique uses a specific oligonucleotide (20–100 bp) to generate a unique DNA base change in the plant genome. The cell repair system recognizes the single base mismatch between its own DNA and that of the repair template.

Genotyping-by-sequencing (GBS) allows for simultaneous marker discovery and genotyping approaches, and delivers many benefits, including the availability of flanking DNA sequence information, high-sample throughput, and high resolution, among others [180]. Next-generation next-generation sequencing (NGS) technology has evolved rapidly, which has allowed the cost of DNA sequencing to be significantly reduced, making GBS increasingly accessible to a greater diversity of species with large genomes [181]. GBS has significantly increased the availability and applicability of molecular markers for crop improvement [182]. Candidate single nucleotide polymorphisms (SNPs) identified by GBS may be linked with desired traits with the help of genome-wide association study (GWAS) and/or QTL mapping [92]. The identified SNPs are used in marker-assisted breeding for both to track gene introgression or even to validate trait-linked haplotypes in important agricultural plants (Figure 3a).

GBS provides a rapid and low-cost tool to genotype breeding populations, allowing plant breeders to implement GWAS, genomic diversity studies, genetic linkage analysis, molecular marker discovery, genomic selection (GS), and epigenetic variations under large-scale plant breeding programs [92,182,183,184,185,186,187,188,189,190,191,192,193,194,195,196,197,198,199,200,201,202].

There are some variants of the original GBS protocol [180]. Essentially, to prepare GBS libraries for NGS, genomic DNA obtained from tissues of parents and lines under study is digested with the restriction enzyme ApeKI, which is an enzyme with frequent cutting ability (although recent reports use other enzymes and even a combination of different enzymes) [180,183,185,189,203]. The fragmented DNA is ligated with adaptors 1 (barcode adaptor) and 2 (common adaptor), both containing the corresponding overhang ends for the used restriction enzyme(s). A compatible set of 96 barcode adaptors (from 4 to 8 bp length) were used in the original protocol. Sets of ligation products of digested DNA samples (up to 96), each with a different barcode adapter, are pooled and cleaned up to eliminate unincorporated adapters. To increase the fragment pool, a PCR was performed using primers with compatible sites to the ligated adapters. After clean up and evaluation of the resulting fragment sizes, the PCR products are used for single-end or pair-end sequencing using NGS. Bioinformatic analysis of the resulting sequences allows for mapping and SNPs validation. Although the use of the GBS strategy is growing in use, the intrinsic error rate of the sequencing process and the low depth of sequencing are associated with the two major drawbacks of this approach [204].

The recent high increase in new plant genomes sequenced allows for the comparative analysis of numerous related individuals. There is a high degree of genomic variation, ranging from SNPs to large polymorphisms known as structural variations (SV), which led to the realization that unique reference genomes do not represent the species diversity, thus leading to the pangenome concept. Pangenomes represent the genomic diversity of a given species and include core genes (common genes within all the individuals of the species), as well as variable genes (absent in some individuals) [205,206,207] (Figure 3b). Abiotic- and biotic-responsive genes are frequently enriched within variable gene groups [208]. Moreover, the use of accessions of all available species of a given genus for the development of a more comprehensive and complete pangenome is now known as the super-pangenome [209] (Figure 3b). Thus, the use of pangenomic and super-pangenome data can be used for developing markers for GWAS and gene discovery to support robust plant breeding programs to achieve abiotic stress tolerance or another desired trait [208,210,211,212,213,214,215].

On the other hand, a tremendous amount of transcriptomic data also continues to accumulate, which could be used to identify genes with potential for plant breeding applications. In pan-transcriptome analyses, a large number of different transcriptome accessions made from varieties of a species or members of a genus of interest are compared. A pan-transcriptome assembly strategy identified genes for the response to abiotic stress and the synthesis of secondary metabolites among varieties of the *Camellia sinensis* tea plant [216]. In rice, heat stress tolerance genes have been identified by combining data from pan-genomes, pan-transcriptomes, and QTLs [217].

Moreover, mixed strategies of “Omics” and “multi-omics” (including genomics, transcriptomics, metabolomics, proteomics, and phenomics, among others) have been applied to crops to improve abiotic stress tolerance [218,219,220,221,222,223,224,225,226,227,228,229,230,231,232,233,234].

However, understanding the flow of biological information underlying complex traits requires a systems biology approach involving the integration of multiple Omics data, modeling, and prediction of cellular functions [235]. It has been proposed that systems biology with multi-omics data integration is important to allow for a holistic understanding of the dynamic system with the different levels of biological organization interacting with the external environment for a phenotypic expression to occur [235].

#### 2.2.2. Zinc Finger Nucleases (ZFNs) and Transcription Activator-Like Effector Nucleases (TALENs)

ZFNs are the predecessors of TALENs, and both are considered the first technologies that started the genome editing revolution era (Figure 3c) [236,237]. Zinc finger nucleases comprise the first generation of tools for genome editing by targeting double-strand breaks (DSBs) using chimerically engineered nucleases. Zinc-finger technology was also the first technology offering efficient and precise genome editing in plants [238,239,240]. The historic record of performance, specificity, and intellectual property rights make ZFNs an interesting genome-editing tool for plant biotechnology [241]. The development of ZFNs technology was possible thanks to the discovery of the functional Cys2-His2 zinc finger domain [242,243,244]. The ZF-binding domain is derived from a human transcription factor and contains four–six Cys2-His2 arrays. Six ZF arrays identify and attach to three nucleotides in the DNA. The α-helices of the ZF DNA-binding domain define which of the three base pairs will be recognized. ZFNs are a combination of a ZF DNA binding domain with an endonuclease, most frequently FokI. Typical ZFNs consist of a minimum of three zinc finger domains, each recognizing three bp [245]. On average, a single functional ZFN pair can be generated per 100-bp DMA sequence, meaning that ZFNs allow for efficient genome editing [246,247]. ZFNs have some advantages when compared to CRISPR technology. ZFNs are able to bind and induce DSBs with high fidelity, while CRISPR/Cas system requires a protospacer adjacent motif (PAM) sequence at the target, which limits the number of suitable targets [248,249]. However, despite the effectiveness of ZFNs, the designing of ZFNs, which perfectly match a specific DNA sequence, is still a strong labor-intensive and costly issue [250].

The TALEN strategy was the first genome-editing tool that saved a human life [251] and also allowed the generation of the first genome-edited crop into the market [252]. TALENs are constituted by a specific DNA binding domain (which is freely designable) and a nuclease [253]. TALENs work as molecular scissors, inducing DSBs to the DNA at a specific site [245]. The binding domain attaches to a specific DNA sequence, and the nuclease domain creates DSBs. The induced DSBs are further repaired by non-homologous end joining (NHEJ) or homologous recombination (HR) [150]. When NHEJ participates in the DSB reparation, this usually disrupts the gene function due to the generation of small insertions or deletions at the DNA breakpoint [245,254]. In HR, the DNA region flanking the DSB is replaced with a repair template or similar sequence [253,255].

TALEs were first identified in the plant pathogen Xanthomonas, where they are delivered to host cells to reprogram the plant transcriptome, suppress immunity, support pathogen growth, and promote the development of disease [256,257]. The Xanthomonas TAL effectors bind to specific sites of the plant host DNA and activate the expression of genes thought to be beneficial to the pathogen’s growth and dissemination [256,258,259] TALEs contain a central DNA-binding repeat domain (CDR) that addresses the DNA binding specificity through one repeat to one base pair correspondence [260,261]. The CDR contains tandem repeats of 34 amino acid residues, and each CDR repeat binds to one nucleotide in the target DNA site. The two amino acids that define the DNA specificity of a TALE or TALEN are located at positions 12 and 13 of each repeat, named the repeat-variable di-residue (RVD) [260,262]. The specificities of all possible RVD have been decoded, revealing very specific ones recognizing only a single nucleotide as well as more flexible ones tolerating two, three, or all four nucleotides [256]. DNA binding specificities of TALEs and TALEN can be changed at will, through a repeat re-arrangement. Using this feature, TALEs can be engineered to bind any desired DNA sequence [263]. The TALE domain was first used as a fusion protein to address the nuclease activity of the enzyme FokI [264,265,266,267]. The nuclease FokI works as a dimer, so TALENs are designed in pairs that bind opposing DNA target sites separated by a 12–21 bp spacer [268]. This configuration allows the FokI monomers to come together to generate a DSB. In addition to nucleases, TALEs have been used to fuse repressors or activator domains in order to address, respectively, gene knockdown or gene activation; to fuse transposases, recombinases, reporter proteins, or histone modifiers such as methytransferases and DNA-cytosine demethylases to conduct epigenetics research [255,268,269]. Moreover, TALENs have been used to achieve multiplex genome-editing (MGE), for example, using a single TALEN pair to edit three homoeoalleles encoding mildew resistance locus proteins in hexaploid bread wheat [270,271]. In addition to nuclear genome editing mediated by TALENs, this technology has been successfully used to target and edit mitochondrial and chloroplast sequences (mitoTALENs and cpTALEN, respectively) [272,273,274,275,276] (Figure 2f,g).

The generation of plasmids for TALE expression can be a slightly complex issue due to their repetitive nature, although the design is easier than for ZFNs [277] However, the development of high-throughput cloning methods such as the Golden Gate, GoldenBraid, Gibson assembly, Gateway assembly, or chaining cloning, has accelerated the way in which the mass editing of genomes is carried out [278]. Several toolkits for the construction of tailor-made TALEs with custom DNA specificity are available [279,280,281,282,283,284].

#### 2.2.3. Clustered Regularly Interspaced Short Palindromic Repeats (CRISPR)

The discovery that prokaryotes also possess mechanisms that confer adaptive and heritable immunity constitutes one of the greatest advances in biology in the last two decades. The history of CRISPR began with the description of a series of short direct repeats interspaced with short sequences in the genome of *Escherichia coli* [285]. The prokaryotic CRISPR adaptive immune system (existing in 50% of bacterial genomes and 90% of archaeal) is able to store memories of past phage infections and, when suffering a new infection, utilize RNA-guided nucleases, CRISPR-associated (Cas enzyme). This mechanism induces the silencing of phages and other mobile genetic elements, such as plasmids and transposons, in a sequence-specific manner. Twenty-five years later, the tool for genome editing was developed using the Cas9 endonuclease [248]. Almost simultaneously, the first examples of the CRISPR-Cas 9 system were reported in humans and other eukaryotes [286,287,288,289]. The first successful applications of CRISPR/Cas technology in plants were reported in tobacco, wheat, rice, sorghum, and Arabidopsis, as well as the first discussions about its application in crops [290,291,292,293,294,295,296,297]. The importance of CRISPR-Cas technology was recently recognized with the Nobel Prize in Chemistry in 2020 to Emmanuelle Charpentier and Jennifer Doudna for their groundbreaking work on the CRISPR system [248].

To understand the applications and limits of the CRISPR-Cas technology, it is important to know the composition and role of prokaryotes. The CRISPR/Cas immunity process can be divided into the following three stages: adaptation, crRNA maturation, and interference [298,299,300]. During adaptation, a complex formed by the nucleases Cas1 and Cas2 recognizes and selects a fragment of the foreign DNA and integrates it into the host’s CRISPR array. The crRNA maturation involves the generation of a long pre-crRNA as a result of the transcription of the CRISPR array, which is further fragmented by Cas proteins (or cellular endogenous RNases) releasing several individual mature crRNAs. Then, the mature crRNAs guide Cas nucleases to their target foreign DNA (the interference stage). The Cas proteins cleave the foreign nucleic acid after crRNA recognizes the target sequence by sequence complementation and binding (Figure 3d). Regarding the structural genomic composition of the CRISPR-Cas locus, this is constituted by the *cas* operon (expressing several Cas nucleases) and the CRISPR repeat-spacer-array (which contains all the spacers that store the memory of previous phage-mediated infections); in the interference mediated by the nuclease Cas9, the transcription of the tracrRNA (trans-acting CRISPR RNA) from a neighbor locus is also required [301]. Taking into account the variety of *cas* genes and the biology of the interference complex, CRISPR-Cas systems have been assigned to two classes, which are subdivided into six types and various subtypes that each possess signature *cas* genes. Class 1 CRISPR-Cas systems (types I, III, and IV) employ multi-Cas protein complexes for interference (Cas5, Cas7, Cas8, Cas11, among others). In contrast, in class 2 systems (types II, V, and VI), interference is achieved by a single effector protein, such as Cas9, Cas12a, or Cas13 (Rnase) [300,302,303,304,305,306,307,308]. However, more weapons have evolved in this molecular war. Bacteriophages also harbor a battery of anti-CRISPR (Acr) proteins that tend to suppress the CRISPR-Cas immunity of the infected bacteria. The Acr proteins act at different levels, inhibiting the crRNA-guided DNA binding and priming adaptation or blocking the cleavage of phage DNA [309,310].

The CRISPR-Cas9 system is the most widely adopted tool by the scientific community for genetic engineering. It is constituted by Cas9 nuclease, tracrRNA, and crRNA. The Cas9 protein is a crRNA-dependent endonuclease, which is analogous in shape to a bilobed jaw and contains one nuclease (NUC) lobe, and one recognition (REC) lobe. The Cas9 NUC lobe contains two unrelated nuclease domains (RuvC and HNH), which are responsible for the cleavage of the displaced (non-target) and the target DNA strands, respectively, in the crRNA-target DNA complex [248,304,311]. The NUC lobe of Cas9 also contains a PI domain, which can recognize the PAM sequence on the non-complementary strand. The PAM is a short sequence motif (usually 2–6 base pairs in length) adjacent to the crRNA-targeted sequence on the invading DNA and plays an essential role in the stages of interference and adaptation [312]. To be able to cut, Cas nuclease requires a PAM, which is usually located 3–4 nucleotides downstream from the cut site. For sequence-specific silencing, crRNA and tracrRNA participate in target recognition [298,299]. The 5′-terminal sequence of each crRNA is complementary to the sequence of the target site, while the crRNA 3′-terminal sequence can create complexes with tracrRNA and Cas9. The Cas9-tracrRNA-crRNA complex is essential for the identification and binding of Cas9 on the proper target sites and specific cleavage, resulting in a DSB on the processed DNA. Repair of these DSBs can lead to gene disruption if the break is repaired by a deleterious event resulting from a classical nonhomologous end-joining reaction (C-NHEJ), an alternative end-joining reaction (alt-EJ, also called microhomology-mediated end-joining, MMEJ), or a single-strand annealing reaction (SSA) [313]. Alternatively, in the presence of a homologous donor DNA template, these DSBs can be repaired via a homology-directed repair (HDR) pathway, leading to accurate gene replacement [313,314].

For its practical use in different organisms, the CRISPR-Cas9 systems have been adjusted. The artificial CRISPR-Cas9 system includes a synthetic RNA chimera created by fusing crRNA with tracrRNA (single guide RNA, or sgRNA), which is functional and comparable in efficiency to the crRNA and tracrRNA complex (Figure 3). Consequently, the number of components was brought down to only two, Cas9 and sgRNA [248]. The 5’end sequence of the sgRNA guide provides DNA target specificity. Consequently, it is possible to design sgRNAs with different target selectivity by changing the 5’end sequence of the sgRNA guide. The length of a canonical guide sequence is 20 bp. Accordingly, the length of a DNA target is also 20 bp, followed by a PAM sequence that contains the consensus NGG sequence. Another innovation to implement the CRISPR-Cas9 systems in plants was the use of a plant codon-optimized version of Cas9 from Streptococcus pyogenes, although a previously reported human codon-optimized version of Cas9 has also been used successfully [290,315,316,317].

In summary, the use of CRISPR-Cas9 in plants involves a four-step process. First, design the gRNA sequence for the selected target genome region, including a sequence of 20 bp followed by PAM (NGG). Next, assemble a codon-optimized Cas9/sgRNA construct (s) with a plant RNA polymerase III promoter (AtU6 or TaU6 or OsU6 or OsU3, or a species-specific RNA polymerase III promoter). Third, the stable or transient transformation of sgRNA and Cas9 into plant tissues (via Agrobacterium, biolistic, or protoplast) [140,318,319]. Recently, virus-based vectors were also proposed for plant delivery of CRISPR-Cas9 [320]. The last step, screening for mutant or transformed plants by PCR genotyping and confirmation by sequencing. Several tools for the design of CRISPR-Cas9 components are available online [321,322].

Remarkably, close to twenty CRISPR-Cas system variants have been developed to edit the genomes at different levels, which represents a promising battery of CRISPR tools for plant genome editing, allowing not only for DSBs but also for base editing, gene repression or activation, chromatin topology or imaging, and epigenome editing, among other applications (Table 1 and Figure 3d) [300,323]. First, CRISPR-Cas12a; the system CRISPR-Cas12a produces DBSs with sticky ends (4–5 nucleotide overhangs), which can be used for DNA precise repair with the use of a donor repair template (DRT), which is inserted by the action of the HDR pathway [324,325]. Second, DNA and RNA base editing; base editing uses CRISPR-Cas machinery to convert one single base to another in a programmable manner, without the generation of DSBs or DRT participation [326,327]. At least the following three classes of base editor systems are known: adenine base editors (ABEs), cytidine base editors (CBEs), and RNA editing (REPAIR) systems. CBEs contain the mutant variant nCas9 (D10A mutation) or SpCAs9-NG variant fused with cytidine deaminase (which mediates G-C to A-T conversion in the targeted DNA strand) [326,328,329]. The nCas9 mutant has nickase activity while still being capable of binding sgRNA [330,331]. ABEs are composed of an adenine deaminase fused to nCas9 for A-T to G-C base conversion [327,332,333]. In both cases, CBE and ABE, the further DNA mismatch repair mechanism and DNA replication allow for the base change fixation. In addition to DNA editors, RNA base editors enable single base substitutions at the RNA level. As an example, the REPAIR system uses a deaminase or an adenosine deaminase (acting on RNA) fused to catalytically dead dCas13b [334,335,336,337]. This allows for programmable RNA editing, changing A to I, which is treated as guanine during translation. Moreover, the RESCUE systems use a dCas13b catalytically dead endonuclease domain fused to engineered ADR2DD for C to U base replacement on RNA [337,338]. Third, Prime editing; the prime editors are bipartite systems, containing a prime editing guide (pegRNA) and a fusion protein (M-MLV reverse transcriptase fused to nickase nCas9 (H840A) [339,340]. The pegRNA is a modified version of a sgRNA, additionally containing a primer binding site (PBS) sequence harboring a template sequence for reverse transcriptase (RT); this PBS contains new or edited genetic information. The pegRNA guides the protein fusion to the target, and the PBS region binds to the induced nicked DNA strand and initiates the reverse transcription of the template sequence that contains the desired edit or new genetic information [330]. The DNA repair mechanisms allow for the fixation of the edition. Fourth, Epigenome editing; there have been developed epigenome editing systems, which alter the methylation profile of DNA (CRISPR-SunTag system) or RNA (m6A editing system), as desired. The CRISPR-SunTag system (dCAs9-SunTag-TETcd demethylase or dCas9-SunTag-DRM methyltransferase) enables target DNA demethylation and methylation, respectively, to repress or activate gene expression [341,342,343]. Fifth, tissue culture-free genome editing; this method generates genome-edited plants by removing the meristems and inoculating the injured cut portion of the plant with Agrobacterium harboring developmental regulators (DRs) and CRISPR constructs, inducing the production of new meristems. Eventually, new genome-edited shoots form and the changes are transmitted to the next generation [344]. Among the DRs used are WUSCHEL (WUS), SHOOT MERISTEMLESS (STM), and MONOPTEROS (MP), whose action dictates, in part, the meristems’ identity. Sixth, CRISPR-IGE (inducible genome editing); at least the following two inducible systems have been used to manage the expression of Cas9: the XVE-inducible for 17-β-estradiol [345] and the HS-CRISPR inducible by heat shock [346]. In addition, a successful combination of CRISPR-TSKO with CRISPR-IGE has been reported [345]. Seventh, CRISPR-TSKO (CRISPR-based tissue-specific knockout); this system allows for efficient mutagenesis in a cell-, tissue-, or organ-specific manner by addressing Cas9 expression under the control of a specified cell or tissue type promoter [347]. This allows for temporal and spatial regulation of edited genes in plants. Eighth, CRISPR-SKIP; this strategy of base editing uses CBEs to mutate G at the end of introns, just in the boundaries of exon-intron, altering the resulting RNA splicing. Thus, altering a single base can lead to exon skipping into mature transcripts, removing exons permanently [348]. Ninth, CRISPR Start-Loss (CRISPR-SL); this method can abolish targeted gene expression by disturbing the start codon (ATG), using both CBEs and ABEs to convert ATG into ATA, ACG, or GTG [332,333,349]. Tenth, DNA-free genome editing; this innovation uses the delivery of pre-assembled gRNA/Cas9 ribonucleoproteins (RNPs), which facilitates gene editing without the use of transgenes, avoiding their integration in the genome [350,351,352,353,354,355].

Recently, also a plant negative-strand RNA virus-based vector was designed for in planta delivery of the entire CRISPR-Cas9 cassette to achieve single, multiplex mutagenesis and chromosomal deletions in tobacco; the vector can be readily eliminated from mutated plants during regeneration, allowing for DNA-free genome editing [319]. Eleventh, CRISPR-STOP is a method that creates stop codons using base editors (without DSB events), consequently provoking gene silencing. The CBEs utilized generate UAA (ochre), UAG (amber), and UGA (opal) stop codons by targeting the cytosine in the coding strand of the target harboring CAA (glutamine), CAG (glutamine), and CGA (arginine) [356]. Twelfth, CRISPR-PASS; this method eliminates premature stop codons (PTCs) by disrupting stop codons such as TAA, TAG, or TGA. For this, CRISPR-PASS uses ABEs to convert such codons into TGG (tryptophan) [357]. Interestingly, the strategy of inactivating PTCs in key gene transcripts is a promising way to improve desired characteristics for plant crops.

MGE supported by CRISPR-Cas confers to scientists the capacity to decode complex biological problems by editing several genes simultaneously. This allows the knockout of multiple genes at once to engineering-efficient plant metabolic pathways, to manipulate the transcriptional regulation of a group of genes, to achieve chromosomal segment restructuring, or multiplex base alterations, among other applications [271].

As mentioned, the engineering of Cas proteins provides new variants with the potential to generate novel applications for genome editing. Nature is also an excellent source of new forms of Cas proteins. A recent report showed the isolation of a large number of new phages from a wide range of ecosystems’ sampling collections [358], achieving the identification of ubiquitous huge bacteriophages with a surprising prevalence of CRISPR-Cas systems encoded in their genomes. The clade Biggiephage harbors a member of the Cas family with about half the size of Cas9 or Cas12 (140 kD), which is known as Cas (Cas12j). The 70 kD Cas harbors an individual active site for the following steps: crRNA processing and the cutting of target nucleic acids guided by a crRNA. This hypercompact system contains expanded target recognition capabilities in comparison with other Cas proteins and is active in vitro and in human and plant cells [359]. Such compact Cas proteins could be engineered to search for new functionalities, thus expanding the toolbox for genome editing. The small size of Cas, coupled with their minimal PAM requirement will be particularly advantageous for both vector-based delivery into cells and a wider range of targetable genomic sequences, providing a new component to the CRISPR-Cas toolbox [359].

Finally, the use of the CRISPR-Cas system for gene editing in chloroplasts expands the alternatives for genetic improvement (Figure 2g). Delivery of the plasmids in a targeted manner to the chloroplast can be accomplished using biolistics and carbon nanotubes [360,361].

### 2.2.4. Oligonucleotide-Directed Mutagenesis (ODM)

The ODM is now being revisited as a suitable technique to make directed genome editing in plants. ODM allows the generation of custom-made SNPs in the target genome, which represents a “game-changing” in the plant breeding area. One interesting advantage of ODM is that the resulting genome-edited plants are considered non-transgenic [362,363], opening the possibility to apply this technique in plant crops without costly and long GMO regulatory procedures. Initially, ODM was successfully used for genome editing in bacterial, yeast, and mammalian systems [364,365,366]. In plants, ODM started to be used at the end of the nineties [367,368,369,370,371].

The mode of action for ODM in eukaryotic systems has been determined in mammalian and plant models. An oligonucleotide with homology to the target sequence but containing a mismatch is delivered across the cell membrane, travels through the cytoplasm, and finally passes the nuclear membrane where it anneals to the nuclear DNA target sequence [372]. In addition, chimeric RNA/DNA can also be used in plants for ODM [369]. ODM requires the use of oligonucleotides (between 20 and 100 nucleotides in length) designed to be identical to the target except for one or a few altered nucleotides corresponding to the intended mutations [371,373]. The endogenous mismatch repair system is involved in the incorporation of the (oligo-directed) nucleotide mismatch [365,367,374] (Figure 3e). Importantly, it is critical to have high transfection efficiencies to ensure the oligonucleotide is delivered into as many cells as possible to maximize DNA conversion efficiencies; otherwise, the oligonucleotide-mediated conversion rate could be as low as that reached by the spontaneous mutation rate [375]. ODM involves the following two-step process to insert the mutation: first the oligonucleotide annealing to the sequence target, and second, the reparation of the mismatch by the cell´s repair machinery. Although in plants, the oligonucleotide does not integrate into their genome, it serves to guide the cellular repair system to the target site. Its inability to integrate is due to the following two reasons: the activity of endogenous nucleases and other oligonucleotide degrading enzymes, and the 5’ and 3’ end modifications in oligos, which prevent DNA ligation [362]. On the other hand, in prokaryotes, the ODM system requires the action of mustS and recA to incorporate the ODM-induced mismatch into the genome [362].

The molecular basis of many important agronomical traits relies on small genetic differences, or SNPs, present in critical genes. A classic example of the ability of ODM to incorporate SNPs into the plant genome is the manipulation of the acetohydroxyacid synthase (AHAS) genes of tobacco and maize [367,369,376]. ODM-induced editing on AHAS confers resistance to the herbicide chlorsulfuron, which is a specific AHAS-inhibitor herbicide by blocking the synthesis of the branching amino acids valine, leucine, and isoleucine [377]. A sulfonylurea herbicide-tolerant canola variety (SU Canola^TM^) was the first commercialized ODM genome-edited (Ged) crop, which was obtained by pit mutation in AHAS (also known as ALS, acetolactate synthase gene) [362,378,379]. The SU Canola^TM^ was considered non-genetically modified (non-GM), classified as “non-regulated”, and launched in the USA in 2015 [380] and in Canada in 2017 [135,372,381].

Finally, oligos have also been used to generate mutagenesis in Arabidopsis chloroplast lysates [382]. It would be interesting to explore the possibility of using ODM for the modification of chloroplast genes (Figure 2g) using carbon nanotubes [360] or other types of nanoparticles for the delivery of oligos in this organelle.

## 3. Crops

### 3.1. Model and Non-Model Plants

The use of model plants such as Arabidopsis, maize [383], bryophytes such as *Physcomitrium patens* (formerly *Physcomitrella patens*) [384], halophytes such as *Mesembryanthemum cristallinum* [385], *Thellungiella halophila*, *Aeluropus littoralis* [386], resurrection plants such as *Craterostigma plantagineum* [387], *Selaginella lepidophylla* [388], and *Pseudocrossidium replicatum* [389], as well as important crops including barley, potato, soybean, common bean [390], tomato [391], among others, have established the bases of plant abiotic stress biochemical, genetic, molecular, and physiological responses. Genome sequencing, gene annotation, functional Omics, and validation of stress tolerance identified genes from model plants as well as from major crops have been vital. Although multiple important successful efforts to improve abiotic stress tolerance through conventional breeding (induced mutation, inter-generic and inter-specific crosses), molecular and biotechnological approaches (QTL, marker-assisted selection, haplotype analysis, genome-wide association studies–GWAS, genetic engineering, and genome and epigenome editing technology), as well as the use of effective microbes, have been reported, the search for stress tolerance can only be reached considering the physiology, ecology, and breeding of individual plant species under realistic field conditions. Although not described in this review, improvement of abiotic stress tolerance in several important crops such as barley [392,393,394,395], sorghum [396], potato [397,398,399,400,401], leguminous [400,402,403,404,405,406,407], and horticultural [400,408,409,410] plant species has been described.

In the following sections, selected recent reports that describe efforts to obtain abiotic stress-tolerant plants, though mentioned techniques are briefly described, focusing on the three main cereal crops, rice, wheat, and corn, comprising around 75% of grain production worldwide.

### 3.2. Rice (Oryza sativa *L*.)

Rice is one of the major abiotic stress-sensitive sources of food. Backcrossing and induced mutation methods have led to the development of rice drought-tolerant varieties that include important traits such as root architectural plasticity [411,412,413,414,415,416]. Gamma rays irradiated rice varieties or landraces have rendered at least 16 drought-tolerant lines, for example, MK-D-2, MK-D-3, MR219-9, and MR219-4 [411,412,413,414]. Moreover, QTLs and marker-assisted selection (MAS) strategy have been conducted to the identification of hundreds of abiotic stress tolerance QTLs (extensively reviewed by Choudhary, et al., 2019 [417]), including drought tolerance (i.e., deep rooting *DRO1* QTL) [94,418,419,420,421,422], tolerance to salinity (i.e., *Saltol* QTL) [423,424,425,426,427,428], heat (i.e., QTLs thermotolerance *TT1*, spikelet fertility and pollen shedding *qPSL^ht^4.1*, spikelet sterility *qSTIPSS9.1*) [428,429,430,431,432], cold [433] (Table 2), and flooding or submergence tolerance (such as *SUB1*, *qTIL1*, *qTIL12*, *qNEI12*, *qLEI12* QTLs) [425,428,434,435,436,437,438,439]. Moreover, the combination of multiple stress tolerance (IR64-Sub1 with drought-tolerant lines, UKM5 and UKM91 that contain the qDTYs, viz. qDTY12.1 and qDTY3.1 drought yield QTL loci) [440] and resistance to biotic and abiotic stress [441] has been identified in this crop. It is worth noting that root architectural plasticity QTLs are closely linked to drought and submergence adaptation in rice [416]. Haplotype analysis combined with GWAS led to the identification of the *SEMIDWARF1* gene involved in rice adaptation to flooding [442] (Table 2). Recently, GWAS identified eight cold-tolerance-related genetic loci in this crop, including one locus (*LOC_Os10g34840*) whose cold-tolerant allele is present in most temperate japonica accessions (80%) [443] (Table 2). GWAS in rice has also found numerous markers for tolerance to salinity (*OSMADS31*, *OSHAK11*, *AGO*, *OsPINis*, *Germin family proteins SAP*, *ZIFL*), and flooding, among other traits [444,445].

Omics approaches have led to the identification of hundreds of abiotic stress-responsive genes, and many of them have led to improved stress-tolerant phenotypes when overexpressed (or suppressed). Molecular approaches to improve rice tolerance to several abiotic stresses have been comprehensively reviewed [94,422,470,471,472,473,474,475,476]. Genes encoding enzymes involved in the synthesis of osmolytes and other protectants that improve tolerance to one or several abiotic stresses (drought, oxidative, cold, salinity) in rice plants include functions for arginine decarboxylase (ADC), polyamine synthesis (ADC), abscisic acid metabolism (DSM2), amino acid metabolism (*OsOAT*), reactive oxygen species (ROS) scavenging (*OsSRO1c*), protoporphyrinogen oxidase (*PPO*), trehalose synthesis (*OsTPS1*, *TPSP),* and proline synthesis (*P5CS*). Moreover, proteins encoding for late embryogenesis abundant (LEA) proteins (*HVA1*, *OsLEA3-1*, *OsLEA3-2*), regulatory genes coding for transcription factors (*ABF3*, *AP37*, *OsbZIP23*, *OsbZIP72*, *OsbZIP73*, *OsbZIP42*, *OsbZIP46*, *OsbZIP66*, *SAPK6*, *OsFTL10*, *OsMYB6*, *OsMYB48-1*, *ZAT6*, *SNAC1*, *ONAC045*, *ONAC5/6/9, ONAC10*, *ONAC14*, *EcNAC67*, *IDS1*, *OsPIL1*, *OsERF115/AP2EREB110*, *DREB1A*, *OsDREB1*, *DREB2*, *HvCBF4*, *EDT1*, *OsWRKY11*, *TaWRKY32*, *OsTZF5*), harpin protein (*Hrf1*), jasmonate and ethylene-responsive factor 1 (*JERF1*), ethylene-responsive factor 1 (*TSRF1*), RING finger protein (*OsCOIN*), stress/zinc finger protein (*OsiSAP8*), protein degradation (E3 ubiquitin ligase *OsSDIR1*, *OsPUB67*), nucleolin (*OsNUC1-S*), among others, have lead to increased stress tolerance [94,473,475,477,478,479,480,481,482]. In addition, photosynthetic-related genes, such as the PcCFR gene, coding for a salt-tolerant chloroplastic fructose 1,6-bisphosphatase, transgenic overexpressing rice performed better under salinity, drought, and cold stress [446] (Table 2), as well as over-expression of C4 photosynthetic genes (phosphoenolpyruvate carboxylase enzyme encoding *PEPC* gene, pyruvate phosphate dikinase enzyme encoding *PPDK* gene, NADP-dependent malic enzyme encoding *NADP-ME* gene [401]. Moreover, overexpression of glycine max ω-3 fatty acid desaturase (GmFAD3A) enhances rice cold tolerance [483]. Regarding rice heat-tolerant lines, overexpression and knockout approaches have been used with promising results using genes encoding for heat shock proteins (*hsp101*, *mtHsp70*, *sHSP17.7*), regulatory proteins or transcription factors (ZFP, OsWRKY11, OsGSK1, OsHsfA2e, Sp17), and other proteins (FAD7, SBPase) [428]). Wild and cultivated rice pan-genome analysis [484], enhances the identification of benefic alleles as targets for abiotic stress tolerance for rice improvement.

GWAS has also identified rice genotype-dependent differential methylation, indicating a role of epigenetic DNA methylation modifications involved in drought stress [485,486]. The exploitation of RNAi technology has been used in rice to increase drought tolerance by silencing C-kinase1 receptor, RING finger E3 ligase OsDSG1, miR170, miR171, miR172; PCF5/PCF8 for cold tolerance [486]. Heterologous expression of salt-tolerance genes from halophytes such as *Suaeda salsa* (*SsNHX1*), *Spartina anglica* (*SaNHX1*), *Puccinellia tenuiflora* (*PtNHA1* and *PutNHX*), *Spartina alterniflora* (*SaVHAc1*, *SaSRP3-1*), *Atriplex hortensis*, *Suaeda maritime* and *Suaeda liaotungensis* (*Badh*), *Porteresia coarctata* (*PcINO1*), and *Avicennia marina* (*Sod1*) improved salt tolerance in transgenic rice [386].

Genome editing tools such as CRISPR systems have revolutionized rice breeding. CRISPR/Cas9 gene editing has been shown to improve rice tolerance to cold (*OsPIN5b*, *GS3*, *OsAnn3*, and *OsMYB30*) [447], drought (*OsPYL9*, *OsERA1*, *OsSRL1*, *OsSRL2*, *DST*, *OsmiR535*), salinity and osmotic stress (*OsSAPK2*, *OsRR22*, *OsDST*, *OsmiR535*) [448,449,450,451,476,487], or various abiotic and biotic stresses (*OsMPK5*) [297], among other multi-targeted rice genes [488] (Table 2).

Finally, an effective and promising strategy to increase tolerance to several abiotic stresses is the use of effective microbes applied as inoculants individually or in consortia, which have been shown to improve rice growth and development under drought, salinity, or flooding conditions (reviewed in [77,79,85,489,490,491,492,493,494,495]) (Table 3).

### 3.3. Wheat (Triticum aestivum *L*.)

Several abiotic stresses severely affect wheat growth and yield, especially considering that this crop is mainly cultivated in semiarid and arid regions worldwide where land degradation, water scarcity, and soil salinity cause serious yield losses. Challenging particular complexity is presented by the hexaploid large genome (17 Gb and 80% of repetitive sequences) in this crop [504].

Wheat has a history of systematic breeding over 100 years; accordingly, huge wheat germplasm has been developed that includes more than 800,000 local landraces, domesticated wheat species, breeding derived, and synthetic accessions (obtained by interspecific hybridization techniques) that contain drought-, heat-, cold-, salinity-, and waterlogging-tolerant genetic resources [505]. Among the landraces, varieties, and breeding-derived lines that exhibit drought tolerance are Aka Komugi (source of the dwarfing *Rht8c* allele), Creole, *Triticum boeoticum*, Kauz, Ningchun 47, Nesser, NI-5439, WH-1021 and HD-2733, Alvd//Aldan/Ias58*2/3/Gaspard, Pavon 76, Chakwal-86, drought-tolerant 1B/1R chromosome translocation wheat genotypes, among others [505,506,507,508]. Wheat drought and salt stress tolerance improvements have been obtained by the introgression of traits from the wild relatives *Agropyron elongatum* and *Aegilops umbellulata* [509,510]. Mutation breeding (gamma radiation) has led to the obtaining of multiple drought-tolerant wheat lines [511,512]. Induced mutation has also been used to improve wheat salinity tolerance (Binagom-1 mutant from L-880 parent cultivar) [513].

Although abiotic stress tolerance is complex and governed by multiple QTLs, improved lines were developed for drought, heat, and salinity tolerance in wheat through molecular breeding [417,514]. Synthetic hexaploid, as well as double haploid (DH) derived wheat lines, have demonstrated improved drought tolerance. Linkage mapping, GWAS, GBS, and QTL meta-analysis have potentiated the identification of wheat QTLs and markers associated with drought tolerance in DH and synthetic hexaploid lines [505]. Traits related to abiotic stress tolerance QTLs include coleoptile length, stomatal movement and density, yield, quality, cell and thylakoid membrane stability, relative water content, flag leaf, ABA, days to anthesis, senescence, root architecture and length, seedling and plant height, the maximum quantum efficiency of photosystem II, and shoot and root Na exclusion (reviewed by Choudhary et al., 2019 and Goel et al., 2020) [417,508]. The use of favorable allele identification by allele-specific markers identified drought adaptation-associated genes encoding functions related to a transcription factor (*Dreb1*), a cell wall invertase (*TaCwi-A1*), and lignin promotion (*COMT-3B*) [515]. Additionally, waterlogging tolerance-associated QTLs have also been identified in wheat [439,516].

Improving wheat drought tolerance through genetic engineering has been reported [517]. Transgenic wheat expressing osmolyte-related genes with improved tolerance to drought (pyrroline carboxylate synthase P5CS), salt and drought (mannitol-1-phosphate dehydrogenase *mtlD*), heat, salt, and drought (betaine aldehyde dehydrogenase BADH, *betaA*) has been successful [396,518,519,520,521,522,523]. In addition, overexpression of transporter proteins (TaFER-5B Ferritin) leads to heat, cold, and drought tolerance in transgenic wheat [452]. Moreover, the expression of chaperons such as Cold shock protein SeCspA, and *HVA1* (LEA), enhanced water deficit tolerance in wheat [524,525]. Expression of other stress-associated proteins such as *AISAP*, *TdPIP2*, and *TaPYL4* enhances drought, osmotic, and salinity stress tolerance (Table 2) [453,454,526].

Overexpression of C4 photosynthetic genes like *PEPC* and *PPDK* improved drought and high-temperature tolerance in wheat transgenic lines and also increased grain yield, root system as well as higher osmolytes and photosynthetic capability [401,455,456,527] (Table 2). Another carbon metabolism-associated gene encoding a fructan exohydrolase (*1-FEH w3*) when overexpressed increases wheat grain yield under drought conditions [528].

Recent reports on transcription factors gene modulation/overexpression in wheat have shown to improve tolerance to several abiotic stresses, mainly drought, salinity, and low temperature; accordingly, successful results have been obtained by overexpression of *SNAC1*, *TaNAC69*, *TaBZR2*, *TaWRKY2*, *AtWRKY30*, *TaERF3*, *AtDREB1A*, *GmDREB1*, *TaDREB3*, *TaCBF5L*, *HaHB4*, *AtHDG11*, *TaSHN1*, *TabZIP2*, *TaNF-YB4* (Table 2) [457,458,473,529,530,531,532,533,534,535].

Other regulatory proteins that belong to poorly attended areas of abiotic stress tolerance are the roles of post-transcriptional and post-translational regulation, which include alternative polyadenylation, alternative splicing, riboswitches, differential RNA stability/decay, specific RNA transport/localization, RNA modification, differential translation, post-translation modifications, protein subcellular localization, stability, and activity [536]. In wheat, an outstanding example of the role of post-translational modification of small ubiquitin-like modifiers (SUMOylation) in abiotic responses has been reported. Overexpression of cysteine protease OVERLY TOLERANT TO SALT-1 (AtOTS1) improved drought tolerance and better growth and physiological traits (increasing photosynthesis and chlorophyll content, and delayed senescence) in wheat [460]. In addition, overexpression of phosphoenolpyruvate carboxylase kinase-related kinase gene (*TaPEPKR2*) enhanced drought, osmotic, and heat stress tolerance in transgenic wheat tolerance phenotype is linked with better root system development (Table 2) [459]. Moreover, transgenic wheat over-expressing the Calcineurin B-like protein-interacting protein kinase *TaCIPK23* gene showed tolerance to drought stress [537]. Identification of wheat stress-tolerant gene alleles/variants for use as targets for manipulation is widened by wheat pan-genome studies [538].

Genome editing tools open new possibilities to make targeted modifications in the wheat genome; in fact, improved drought tolerance has been obtained by the CRISPR-Cas9 system editing *TaDREB2* and *TaERF3* multi-targeted wheat genes [461] (Table 2). In addition, the beneficial effects of effective microbes in mitigating moderate and severe abiotic stress in wheat have been reported (Table 3) [77,79,489,490,494,495,496,497,498,499].

### 3.4. Corn (Zea mays *L*.)

As it occurs in most crops, maize growth and productivity are also severely affected by most abiotic stresses. This cereal crop is the most important in terms of global production. Enhancing maize stress resilience through adaptive strategies is crucial to achieving this goal.

Considerable breeding efforts have identified and utilized allelic variance that confers abiotic stress tolerance in maize. QTLs identification/introgression and marker-assisted selection molecular breeding (QTLian breeding) has also been used to improve drought, waterlogging, heat, cold, and salinity tolerance in maize (extensively reviewed in [201,417,439,539,540,541]). Selected traits for such purposes include cell membrane thermo-stability, germination index, emergence rate, seedling height, leaf firing and temperature, chlorophyll content, low anthesis-silking interval, brace roots, root length, root cortical aerenchyma, shoot and root fresh and dry weight, grain weight, grain yield per plant, kernels per ear, ear length, and reduced kernel abortion, among others [417,541]. Double haploid (DH) technology has been widely and successfully used in maize, where more than 200,000 DH lines have been developed to obtain elite climate-resilient maize cultivars [542].

GWAS has identified gene variants and markers for maize abiotic stress-tolerance improvement [543]. Importantly, root architecture plasticity QTLs as well as genes identified by transcriptomic approaches under water-deficit conditions are closely related, as most of them are specific and most regulated in the cortex of the mature root zone and the elongation zone changes in the root tip, comprising functions associated with cell wall reorganization, allowing continued root growth in water-deficit conditions [416]. These pieces of evidence show clear root-plasticity and stress-tolerance productivity relationships with multiple identified QTLs and promising candidate genes to increase stress tolerance in crops. These findings are in agreement with maize hybrids that show higher root density, have better water use, biomass accumulation, and higher yield potential, associated with heterosis [544]. Attention should be considered on stress-responsive alternative splicing variants, which have been identified in maize to affect root function and structure and cell wall properties; in addition, changes in alternative splicing occur in a tissue-dependent and developmental stage-dependent manner in response to stress [416].

Genomic selection studies identified 77 abiotic stress tolerance SNPs related to ten transcription factors involved in phytohormonal signaling, stomatal closure, photosynthesis, and root development [545]. Abiotic stress tolerance-related genes from local landraces and wild relatives have enormous potential as genetic resources to enhance abiotic stress tolerance in maize. That is the case of *Zea parviglumis* (teosinte) and *Tripsacum*, or waterlogging-tolerant wild maize *Zea nicaraguensis* [201,546,547]. These genetic resources can be significantly broadened by maize pan-genome and pan-transcriptome approaches [548,549,550].

Numerous differentially expressed genes have been identified in water-stressed maize plants, which provide candidate genes for stress tolerance [551,552]. Expressing genes for compatible osmolytes or osmoprotectants such as amino acids and sugars, which assist osmotic adjustment, has been successfully used to improve water use efficiency under stressing and non-stressing conditions, as stated above. Yield improvement was observed when trehalose-6-phosphate phosphatase is expressed in maize ears under both drought and well-watered conditions [33]. On the other hand, bacterial cold shock proteins (CSP_S_) are important bacterial RNA chaperones that maintain RNA stability for bacterial acclimatization to low temperature and drought stress, whose expression in transgenic maize conferred tolerance to drought stress and improved grain yield under water-deficient conditions [463]. Moreover, the expression of CspB protein leads to drought-tolerant transgenic maize (Genuity^®^ DroughtGuard™, MON 87460 event) and other drought-tolerant with herbicide-resistance and/or insect-resistance were developed and successfully used under field conditions (reviewed in [135]). In addition, pyramid heterologous co-overexpression of *betA* (encoding choline dehydrogenase from *Escherichia coli*) and *TsVP* (encoding V-H+ -PPase from *Thellungiella halophila*) resulted in increased glycinebetaine content and H+ -PPase activity, solute accumulation, relative water content (RWC), decreased cell damage, and higher yields under drought stress in transgenic maize plants [462] (Table 2). Heterologous expression of bacterial Vitreoscilla hemoglobin (VHb) increases waterlogging tolerance in transgenic maize due to improvements in root and shoot traits [464]. Another important trait is stomatal density and morphology, which impact CO_2_ uptake and transpiration. These aspects are promising targets to improve water-use efficiency; in this respect, it should be noted that the heterologous overexpression of *AtSDD1* (encoding subtilisin-like protease STOMATAL DENSITY AND DISTRIBUTION1) in maize leads to enhanced drought tolerance by reducing stomatal density [465].

Notably, signaling components involved in abiotic stress response are also key targets for crop improvement, which is the case of mitogen-activated protein kinases (MAPK). Heterologous constitutive expression of NPK1 in maize increased leaf number, photosynthesis rates, and kernel weights under drought stress, leading to improved drought tolerance [553].

Controlling transcription factors has emerged as a promising tool for controlling the expression of multiple stress-responsive genes under multiple stressing actual field conditions. Transcription factor overexpression has been successfully used in maize to enhance abiotic stress tolerance, mainly drought, salinity, and high/low temperature. A common finding is that overexpression of transcription factors confers tolerance to more than one abiotic stress and sometimes also modifies disease resistance [473]. Improved growth and corn yields were reported in transgenic maize-overexpressing *ZmNF-YB2*, under field relatively severe drought conditions [554]. In addition, transgenic maize overexpressing OsMYB55 increased drought and high-temperature tolerance by reducing lipidperoxidation and ROS levels [466]. Waterlogging tolerance has also been improved in maize plants overexpressing ZmERB180 [467] (Table 2).

Top biological regulators such as long non-coding RNA (lncRNAs) have been identified in response to combined abiotic stress (boron and salinity) in a deep RNAseq analysis in the hyper-arid Lluteno maize landrace from the Atacama Desert, where 1710 lncRNAs turned out to be responsive to both stresses’ combination [555]. This set of lncRNAs could represent biomarkers and key targets acting at epigenomic, transcriptional, and post-transcriptional levels in maize.

Epigenetic DNA methylation modification-related genes have been involved in cold-stress tolerant maize, for example, root-specific hypomethylation of the *ZmMI1* gene as well as genome-wide global methylation shift [486]. Moreover, RNAi technology on PDH, POK, MAPK, PLD proteins, and 11 miRNAs has been successfully used to improve drought tolerance in this crop [486].

Improved grain yield under field drought stress has been obtained by the CRISPR-Cas9 system editing the *ARGOS8* maize gene [468]. Maize salinity tolerance has also been obtained by editing *ZmHKT1* using the CRISPR-Cas9 system [469] (Table 2). Multiplexed CRISPR/Cas9-based high-throughput targeted mutagenesis [556], multigene insertion, and chromosomal engineering [557], as well as other CRISPR/Cas applications (heterosis, haploid induction), will boost multi-stress-resilient smart maize and other crops [487].

Effective microbes have also been shown to alleviate salt and drought stress in maize when used as inoculants [77,79,489,490,494,495,500,501,502,503] (Table 3).

## 4. Conclusions and Future Perspectives

The development of climate-resilient cultivars (climate-smart crops) is pivotal to a sustainable way to provide sufficient food and energy supplies in a climate-changing world. Since yield and abiotic stress tolerance traits are usually unlinked, it is hard to select both characters by classic breeding. Thus, omics and site-directed mutagenesis approaches could achieve improving stress tolerance on already high-yield selected lines or simultaneously using these novel techniques.

Some considerations:

- Genome/Epigenome (nuclear and organellar) editing and manipulation of key multi-stress-responsive genes or transcription factors have been shown to confer increased tolerance to multiple stressors;
- Altering expression of organellar DNA damage repair system involved genes can lead to more efficient mutagenesis, genetic diversity enhancement, and tolerance improvement to ROS/oxidative stress;
- Emphasis must be considered on post-transcriptional and post-translational regulators (including the huge diversity of types of lncRNAs and recently discovered glycoRNAs) through the use of multiple omics (PlantOmics) integrating genome-wide associations studies and pan-genomic/pan-transcriptomic strategies;
- Plant phenomics will accelerate plant breeding targeted and successful stress-resilient cultivars and their wild relatives under real field conditions;
- It should be taken advantage of multiple cross-talk signaling among diverse challenging atmospheric and soil abiotic (and biotic) factors such as drought, salinity, nutrient deficiency, soil properties, pollution, metal, submergence, anoxia, heat, low/high temperature, wind, light, UV, CO_2_, methane, N_2_O, O_3_, osmotic, oxidative stress, in energy-(sugars), organ-(aerial, roots), tissue-, and phenology-dependent manner;
- CRISPR/Cas9 multiple gene editing for simultaneous expression of structural and regulatory genes represents a promising strategy in order to develop multi-stress-resilient crops;
- Given the evident role of sugar sensing and signaling in abiotic stress responses (sugar-insensitive Arabidopsis mutants are tolerant to abiotic and salt stress), we believe that sugar signaling pathways are key targets to reducing sugar’s negative feedback effect on photosynthesis, which could lead to abiotic stress tolerant phenotypes and increased yields in crops;
- Undoubtedly, much remains to be discovered and learned from the study of resurrection plants and their associated microbiomes, particularly those tolerant to extreme abiotic stress, i.e., *Bryum argenteum*, *Craterostigma plantagineum*, *Pseudocrossidium replicatum*, *Selaginella lepidophylla*, *Syntrichia* (Tortula) *ruralis*, the Arctic and Antarctic moss *Sanionia uncinata*, desert moss *Syntrichia caninervis*;
- Sustainable management of agricultural water and soil resources;
- Diversification of food supply (nutritional diversity) with local plant species;
- Multi-stress experimentation in the laboratory considering variable intensity and timing and recovery capacities related to photosynthesis and growth parameters;
- The enrichment of the seed and soil microbiomes through the use of microbe-effective-based inoculants undoubtedly contributes to the integrated management of crops to mitigate the effects of the multiple stressors that challenge them.
- The integration of all available molecular tools to develop smart climate crops without yield penalty and with no increase in cultivated land area is absolutely necessary.

## Figures and Tables

**Table 1:** Cas enzymes and CRISPR-Cas tools for plant genome editing.

Cas Enzyme	Tools	Uses	References
Cas9 and Cas9 engineered versions	CRISPR-Cas9	Generates DBS on DNA (blunt end). DNA repair mechanisms provoke frameshift mutations resulting in gene knock-out. Use of more than one sgRNA can induce longer deletions or multiplex gene targeting	[290,314,315]
	DNA-free	Requires delivery of gRNA/Cas9 ribonucleoproteins (RNPs) to editing without transgene integration to the genome	[320,350]
	IGE-XVE	Cas9 expression system inducible by estrogens (17-β-estradiol).	[345]
	IGE-HS	Cas9 expression system inducible by heat shock	[346]
	TSKO	Tissue-specific knockout system. A cell or tissue specific promoter controls the expression of Cas9, provoking spatial regulation of gene editing	[347]
	TSKO + IGE	This combination provides spatial- and temporal-regulated genome editing	[345]
	CBE	Mediates G-C to A-T base conversion in target DNA strand	[328]
	ABE	Induce A-T to G-C base changeover in target DNA strand	[332]
	STEME	Used in a high-throughput manner to modify *cis* regulatory elements and genome wide screening	[333]
	STOP	Facilitates gene silencing by creating stop codon without the need of DBS	[356]
	SMART	Based in the rescue of lethal mutations to quickly assess the efficiency of base editing.	[331]
	SL	Provokes alterations in start codon (ATG to ATA, ACG, or GTG)	[349]
	SKIP	Mutates G at the end of an intron, which can lead to exon skipping into mature transcripts	[348]
	PASS	Convert the three possible PTCs (TAA, TAG, and TGA) into TGG (tryptophan)	[357]
	dCas9-SunTag-TET1cd	Epigenome editing through TET1-cd demethylase, allowing for specific gene up-regulation	[341]
	dCas9-SunTag-DRMcd	Epigenome editing through DRM methyltransferase, enabling specific gene down-regulation	[342]
	Prime editing	Creates new genetic changes (or repairing) at the target DNA without DSB or DRT	[339,340]
Cas12a (former Cpf1)	CRISPR-Cas12a	Cas12a targets T-rich regions of the genome where Cas9 is not suitable to use, facilitates multiplexing, assists for precise DNA repair by exogenous DRT. Cas12a generates staggered ends with 4–5 nucleotide overhangs, which is advantageous for genetic insertions or specificity during NHEJ or HDR. Moreover, Cas12a offers future modifications at the same target site, because it cuts DNA strands distal to the PAM sequence	[324,325]
Cas13 and Cas13 engineered versions	CRISPR-Cas13	Cas13 has ribonuclease activity capable of targeting and cleaving ssRNA. Potential applications in plant virus interference or repression of eukaryotic gene expression	[306,308]
	m6A	RNA epigenome editing. Edits the methylation stage of target transcripts	[343]
	REPAIR	RNA editing. For A to I (G) base substitution at RNA level	[334,337]
	RESCUE	RNA editing. For C to U base replacement at RNA level	[337,338]

**Table 2:** Recent examples of genes used to improve abiotic stress tolerance in the three main cereal crops, rice, wheat, and maize.

Crop	Molecular Strategy	Gene	Improved Stress Tolerance	References
Rice	Haplotype analysis with GWAS	*SEMIDWARF1*	flooding	[442]
	GWAS	*LOC_Os10g34840*	cold	[443]
	QTLs and MAS	*TT1*	heat	[430]
	Overexpression	*PcCFR*	salinity, drought, and cold stress	[446]
	CRISPR	*OsMYB30*	cold	[447]
	CRISPR	*OsPYL9*	drought	[448]
	CRISPR	*OsERA1*	drought	[449]
	CRISPR	*OsRR22*	salinity and osmotic stress	[450]
	CRISPR	*OsDST*	drought, salinity, and osmotic stress	[451]
	CRISPR	*OsMPK5*	various abiotic (and biotic) stresses	[297]
Wheat	Overexpression	*TaFER-5B*	heat, cold, and drought	[452]
	Overexpression	*TaPYL4*	drought	[453]
	Overexpression	*TdPIP2*	salinity and drought	[454]
	Overexpression	*ZmPEPC*	drought and high temperature	[455,456]
	Overexpression	*TaWRKY2*	drought	[457]
	Overexpression	*TaBZR2*	drought	[458]
	Overexpression	*TaPEPKR2*	drought, osmotic, and heat stress	[459]
	Overexpression	*AtOTS1*	drought	[460]
	CRISPR	*TaERF3*	drought	[461]
	CRISPR	*TaDREB2*	drought	[461]
Maize	Overexpression	*betA*	drought	[462]
	Overexpression	*TsVP*	drought	[462]
	Overexpression	CSP_S_	drought	[463]
	Overexpression	TPP	drought	[33]
	Overexpression	*VHb*	waterlogging	[464]
	Overexpression	*SDD1*	drought	[465]
	Overexpression	*OsMYB55*	drought and high temperature	[466]
	Overexpression	*ZmERB180*	waterlogging	[467]
	CRISPR	*ARGOS8*	drought	[468]
	CRISPR	*ZmHKT1*	salinity	[469]

**Table 3:** Examples of effective microbes used to combat abiotic stress in the three main cereal crops, rice, wheat, and maize.

Crop	Rice	Wheat	Maize
Growth promoting rhizobacteria or fungi species/strain	*Acinetobacter lwoffii*	*Acinetobacter* sp.	*Alcaligenes faecalis (AF3)*
	*Arthrobacter defluvii*	*Arthrobacter protophormiae (SA3)*	*Arthrobacter pascens*
	*Azospirillum brasilense AZ39*	*Azospirillum brasilense* Sp245	*A. brasilense*
	*Azotobacter vinellandii* (SRI Az 3)	*A. brasilense NO40*	*Azospirillum lipoferum*
	*Arthrobacter nitroguajacolicus* (YB3 and YB5)	*Azotobacter chrocoocum (E1)*	*Azotobacter* sp.
	*Bacillus haynesii*	*Bacillus amyloliquefaciens 5113*	*Bacillus amyloliquefaciens*
	*Bacillus megaterium* (NBRI 20M)	*Bacillus aquimaris*	*B. licheniformis*
	*Bacillus paralicheniformis*	*B. insolitus*	*Bacillus megaterium*
	*Glutamicibacter* sp. YD01	*Bacillus licheniformis*	*B. subtilis*
	*Jeotgalicoccus huakuii*	*Bacillus pumilus*	*B. thuringiensis*
	*Lysinibacillus fusiformis*	*Bacillus subtilis (LDR2)*	*Bukholderia phytofirmans (psJN)*
	*Oceanobacillus picturae*	*Bacillus thuringiensis* AZP2	*Enterobacter* sp. *(FD17)*
	*Pantoea* sp.	*Dietzia natronolimnaea (STR1)*	*Herbaspirillum* sp.
	*Phyllobacterium brassicacearum*	*Enterobacter ludwigii*	*Klebsiella variicola F2*
	*Pseudomonas jessenii* R62	*Enterobacter* sp.	*Massilia* sp. RK4
	*Pseudomonas pseudoalcaligenes*	*Exiguobacterium aurantiacum*	*Paenibacillus favisporus*
	*Pseudomonas putida*	*Flavobacterium* sp.	*Pantoea* sp.
	*Pseudomonas synxantha* R81	*Klebsiella* sp.	*Pseudomonas aeruginosa (Pa2)*
	*Staphylococcus cohnii*	*Marinobacterium* sp.	*Pseudomonas entomophila*
	*Glomus intraradices*	*Mesorhizobium ciceri (CR-30* and *CR-39)*	*P. fluorescens N3*
	*Glomus coronatum*	*Microbacterium* spp.	*P. fluorescens YX2*
	*Glomus constrictum*	*Paenibacillus polymyxa*	*Pseudomonas monteilii*
	*Glomus claroideum*	*Pantoea* sp.	*P. putida (Q7, GAP-P45, UW4)*
	*Streptomyces* sp. strains	*Pseudomonas aeruginosa*	*Pseudomonas stutzeri*
	*Trichoderma harzianum*	*Pseudomonas fluorescence*	*P. syringae*
		*P. syringae*	*Proteus penneri (Pp1)*
		*Pseudomonas* sp. *(E2)*	*Raoultella planticola YL2*
		*Rhizobium leguminosarum (LR-30)*	*Rhizobium* sp.
		*Rhizobium phaseoli (MR-2)*	*Rhizoglomus intraradices*
		*Serratia* sp.	*Streptomyces* sp.
		*Sinorhizobium* sp.	*Trichoderma atroviride*
		*Stenotrophomonas* sp. strains	
References	[77,79,85,489,490,491,492,493,494,495]	[77,79,489,490,494,495,496,497,498,499]	[77,79,84,489,490,494,495,500,501,502,503]

## Data Availability

Not applicable.
